# The Effect of Temperature on *Anopheles* Mosquito Population Dynamics and the Potential for Malaria Transmission

**DOI:** 10.1371/journal.pone.0079276

**Published:** 2013-11-14

**Authors:** Lindsay M. Beck-Johnson, William A. Nelson, Krijn P. Paaijmans, Andrew F. Read, Matthew B. Thomas, Ottar N. Bjørnstad

**Affiliations:** 1 Center for Infectious Disease Dynamics, The Pennsylvania State University, University Park, Pennsylvania, United States of America; 2 Department of Biology, The Pennsylvania State University, University Park, Pennsylvania, United States of America; 3 Department of Biology, Queen's University, Kingston, Ontario, Canada; 4 Department of Entomology, The Pennsylvania State University, University Park, Pennsylvania, United States of America; 5 Fogarty International Center, National Institutes of Health, Bethesda, Maryland, United States of America; State University of Campinas, Brazil

## Abstract

The parasites that cause malaria depend on *Anopheles* mosquitoes for transmission; because of this, mosquito population dynamics are a key determinant of malaria risk. Development and survival rates of both the *Anopheles* mosquitoes and the *Plasmodium* parasites that cause malaria depend on temperature, making this a potential driver of mosquito population dynamics and malaria transmission. We developed a temperature-dependent, stage-structured delayed differential equation model to better understand how climate determines risk. Including the full mosquito life cycle in the model reveals that the mosquito population abundance is more sensitive to temperature than previously thought because it is strongly influenced by the dynamics of the juvenile mosquito stages whose vital rates are also temperature-dependent. Additionally, the model predicts a peak in abundance of mosquitoes old enough to vector malaria at more accurate temperatures than previous models. Our results point to the importance of incorporating detailed vector biology into models for predicting the risk for vector borne diseases.

## Introduction

Mosquitoes are very efficient vectors of human diseases and are responsible for transmitting some of the most devastating diseases today. For many of these diseases, the age structure and abundance of female adult mosquitoes are key in determining the ability of a mosquito population to vector the disease effectively; malaria is one such disease. Malaria is the most prevalent human vector borne disease, with one half of the world population living in areas where there is risk of infection [1]. Despite the widespread transmission it is still difficult to predict future malaria intensity, particularly in the face of climate change. Because the parasites that cause malaria are so strongly tied to mosquitoes for transmission, malaria incidence will change as the climate changes; however, it is still unclear and a matter of debate how the change(s) in transmission will occur [2]–[10].

Mathematical models of malaria transmission have a long history dating back a century [11]. The classic Ross-MacDonald model has been particularly influential and assumptions made in the model have, in various forms, been included in the majority of malaria models that followed [12]–[14]. The focus of Ross-MacDonald and many subsequent models is the human population, assuming that there is a constant adult mosquito population capable of transmitting parasites. The mosquito lifecycle is generally ignored because eggs, larvae and pupae are not involved in the transmission cycle. This is a useful simplification of the system but unfortunately the results of these models do not predict malaria intensity in most endemic regions [14]. There have been exceptions to this generalization, with some models focusing on the mosquito population, and/or the influence of environmental drivers, such as temperature and rainfall [8], [15]–[20]. Of these models, the ones that explicitly include temperature predict a peak in abundance of vectors at temperatures that are higher than those observed to occur in conjunction with malaria transmission in the field [54]. We propose that a disconnect exists between classic model predictions and observed epidemiology that is caused by mosquito population dynamics that depend on ambient environmental conditions and are strongly influenced by juvenile stage dynamics.

Malaria is caused by *Plasmodium spp.* protozoan parasites. Female *Anopheles* mosquitoes pick up *Plasmodium* parasites in a blood meal taken from an infectious person; blood is required in order to develop eggs. The parasites then go though several developmental stages before they migrate to the mosquito salivary glands. Once in the salivary glands the parasites can be transmitted to a susceptible human host when the mosquito takes another blood meal [21]. The time spent developing in the mosquito is known as the extrinsic incubation period (EIP) and its duration is determined by temperature [7], [12], [22].

Mosquitoes have four main life stages: egg, larva, pupa and adult. The three juvenile stages, egg, larva and pupa, are aquatic. Typically 1 to 10% of the eggs that are laid emerge as adult mosquitoes [23]–[29]. The larval stage is the longest of the three juvenile stages and is the only one that feeds. Previous studies indicate that larvae experience the majority of the effects of density-dependence [30], [31]. Density-dependence is thought to manifest in several different ways depending on species. Increased larval mortality and decreased developmental speed are two of the most commonly measured density-dependent effects on juveniles. Because larval conditions determine adult characteristics, density can play through into the adult stage by changing the number of emerging adults and the size, fecundity and survival of adults [30],[32]–[34].

The EIP is often relatively long compared to the life expectancy of mosquitoes. For this reason, the age structure of a given adult mosquito population is a major determinant of that population's vectorial capacity (the ability of the population to transmit the parasite). The common assumption is that only around 10% of the adult population survives to the epidemiologically relevant age [7], [35]. It is unknown, however, what changes occur in the proportion surviving in response to changes in juvenile population makeup or temperature conditions, on which they are dependent. Even a small shift in the adult age structure can have big consequences in terms of the disease burden.

Both *Anopheles* and *Plasmodium* are sensitive to temperature. Because mosquitoes are ectotherms, each life stage is dependent on temperature in the developmental and mortality rates. The blood meal-egg laying cycle, known as the gonotrophic cycle, in adult females is also dependent on temperature. Interestingly, the temperature-dependencies are not the same among the stages, leading to nonlinearities in population responses to temperature [2], [33], [36]–[38]. Additionally, the optimum temperature for parasite growth does not necessarily correspond to the vector optimum. The effects of temperature on mosquito life history and parasite development have been acknowledged for many years; however, these are rarely included in models used to predict malaria transmission.

We developed a model that begins to take into account the complex, nonlinear temperature relationships present throughout the life cycle, as well as intra-stage competition among larvae. The framework draws on a rich body of previous theory that has been developed for modeling stage-structured invertebrate populations [39]–[43]. The model is comprised of a set of temperature-dependent delayed differential equations (DDE). Temperature is included in all the developmental delays, egg-laying and mortality rates. Using the model, we ask how temperature affects adult mosquito age structure and population densities and thus the potential for disease transmission. The combination of nonlinear temperature-dependencies and within stage density-dependence lead to non-intuitive dynamics that emphasize the potential importance of including vector dynamics in future malaria models. Additionally, the predicted age structure of the adult population points to a greater influence of temperature and juvenile stages than previously thought. Furthermore, our model predicts estimates of the peak temperatures for malaria transmission at temperatures that are more in line with the observed biology than when the classic assumption of a static vector population is used. The ability to predict a peak in potentially infectious mosquitoes that lines up more closely with observed malaria incidence is an important development because it allows for a better understanding of the population drivers and dynamics.

## Materials and Methods

### Model

**Figure 1:**
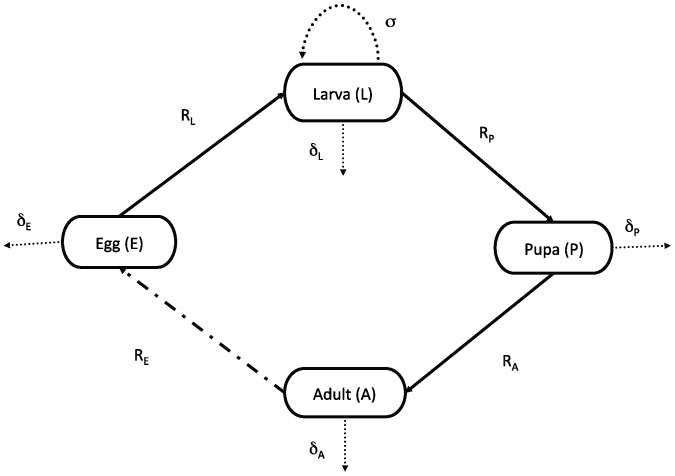
Diagram of the model setup. Each stage experiences temperature-dependent, stage-specific mortality, 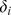 (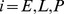 or 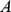). Recruitment into a stage 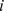 at time 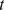 is given by 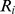 and is also dependent on temperature. Density-dependent mortality is only experienced in the larval stage, 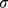.


The framework of the stage-structured, temperature-dependent delayed differential equation (DDE) model reflects details of the mosquito lifecycle (Figure 1). The stage structuring corresponds to the four main life stages in the mosquito life cycle (egg, larva, pupa, and adult) and it allows us to incorporate stage specific life history rates and processes. The stage durations, given by the delays (or lags), are temperature-dependent and allow for biologically realistic developmental times. The temperature-dependence in the delays also allows for the stage duration to change with changing temperatures. We assume that juvenile and adult mosquitoes experience the same temperature. Egg-laying rate, and mortality in all stages, are temperature-dependent, with the larval stage experiencing extra mortality because of the effects of density-dependence. By limiting the effects of density-dependence to larval mortality, the delays present in the model become dependent on temperature alone. The four state equations corresponding to egg (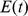), larva (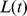), pupa (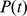) and adult (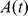) are as follows:
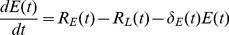
(1)

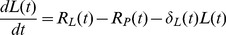
(2)

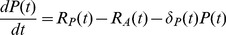
(3)

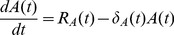
(4)where 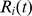 (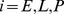, or 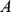) is the recruitment or the flux of individuals into or out of a state and 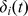 represents the per capita, stage-specific mortality rate. The recruitment into a stage is dependent on the recruitment into the previous stage according to,
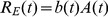
(5)

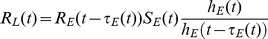
(6)

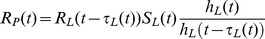
(7)

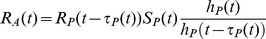
(8)where, 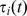 is the duration of stage 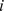 at time 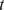. The egg-laying rate (the number of eggs per female per day) is given by 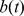 and the temperature-dependent, stage specific development rate is 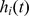. The length of the delay, 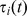, is determined by the temperature-dependent development rate 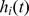 of stage 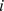 at time 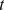. The implication of temperature-dependent time delays in a model is that when temperature changes, delays become variable, which makes analysis more difficult. To alleviate this we re-scaled the model to a physiological time scale so that the delays become fixed [41], [43]. The details of this transformation are presented in the (Text S1 and Text S2). The ratio at the far right hand side of equations 6–8 corrects for any changes in the speed of development within a stage that occur because of any temperature changes during the stage and allows for time varying delays. When temperature is held constant, as in this application of the ratio, the ratio is one and does not impact the recruitment [42]. For the full derivation of this correction, see Nisbet and Gurney [42]. The model works well for fluctuating temperatures and can be driven with stylized or realistic temperature drivers; as a demonstration of this we have included the predicted adult abundance trajectories for a 10°C seasonal fluctuation (Figure S1). However, the systematic exploration of temperature variability is beyond the scope of the current study (see Beck-Johnson et al. *in prep*).

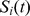
 represents the survival through stage 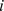 and expands as follows,
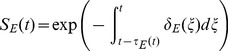
(9)

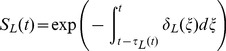
(10)

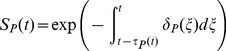
(11)

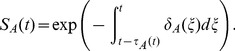
(12)


The stage-specific per capita mortality rate equations (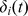) are given by,
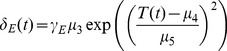
(13)

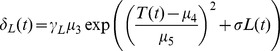
(14)

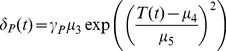
(15)

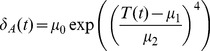
(16)where 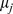 (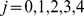, or 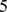) is a scalar and 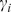 (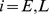, or 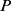) is the proportion of the juvenile life cycle that the eggs, larvae and pupae take up respectively (see Text S1, for derivation). The extra mortality experienced by the larvae because of density-dependence is denoted by 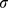. We assume that the larvae are the only stage to experience density-dependent mortality. The Gaussian (and squared Gaussian) functional forms for the temperature-dependence were chosen to fit empirical patterns (see below).

### Parameterization and Data

All parameter values used in the model are based on data from laboratory studies. The functional relationships used in the model were fit to the data using nonlinear least squares optimization. The data that we used to parameterize temperature-dependent mortality rates in the juvenile mosquito stages come from two studies by Bayoh and Lindsay [37], [38]. They monitored mortality at a range of constant temperatures using *An. gambiae* mosquitoes, the main African malaria vector (Figure S2). The data suggest a Gaussian dependence of mortality as expressed in equations 13–15. The adult temperature-dependent mortality data also come from a laboratory study on *An. gambiae* [36], in which mortality rates are followed across a range of temperatures at several humidities (Figure S2). For the purposes of our model, we used the adult mortality data at 60% and 80% humidity, which are both acceptable humidity levels for *An. gambiae* mosquitoes. The functional form that fits adult mortality best is similar to the juvenile stage except that it is raised to the fourth power instead of squared, (equation 16). Parameter values are given in Table 1.

**Table 1:** Parameter Values.

Parameter	Value	Description	Reference
*β*	1.726	fit exponent in development rate function	[17]
*α_I_*	2.87*e*−4	fit scalar in egg to adult development rate function	[17]
*ρ*	0.156	egg-laying rate scalar *E*_0_ *α_G_*	
*μ*_0_	8.86*e*−2	fit scalar in adult mortality rate function	[36]
*μ*_1_	21.211	fit scalar in adult mortality rate function	[36]
*μ*_2_	14.852	fit scalar in adult mortality rate function	[36]
*μ*_3_	2.00*e*−2	fit scalar in juvenile mortality rate function	[37], [38]
*μ*_4_	23.00	fit scalar in juvenile mortality rate function	[37], [38]
*μ*_5_	6.50	fit scalar in juvenile mortality rate function	[37], [38]
*σ_exp_*	1.33*e*−3	fit scalar in density-dependent mortality exponential function (Larvae/Liter)	[31]
*γ_E_*	6	estimate for the proportion of time spent in egg stage	[17]
*γ_L_*	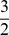	estimate of the proportion of time spent in larval stage	[17]
*γ_P_*	6	estimate for the proportion of time spent in pupal stage	[17]
*α_G_*	1.04*e*−3	fit scalar in gonotrophic cycle rate function	[44]
*E*_0_	150	number of eggs laid per cycle by a single female (observed range 50–300)	[58]–[60]

The data used to parameterize temperature-dependent developmental rates came from multiple laboratory studies on *An. gambiae sensu lato* complied by Depinay et al. [17]. The adult gonotrophic cycle rate, or egg development rate, was parameterized with data from *An. pseudopunctipennis* across different constant temperatures [44] (Figure S2). We fit a power function of the form
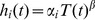
(17)where 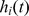 is the development rate through stage 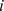 (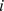 = 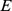, 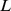, 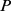, or 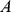). Further information on 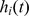 is presented in the (Text S1). Temperature is represented by 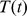 and 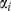 and 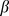 are parameters empirically derived.

The data for parameterizing density-dependent larval mortality came from a laboratory study on four species of mosquito conducted at 27°C; we used data from *An. stephensi* (Figure S3) [31]. Mosquitoes within the *Anopheles* genus appear to respond to density in different ways, through a mixture of increased larval mortality, slowed larval development and feedbacks on adult size, fecundity and survival [30], [31], [33], [34]. In this study we assume increased larval density to increase per capita larval mortality; *An. stephensi* appears to have a relatively strong response in larval mortality to increased density when compared with the response in *An. gambiae* [31]. The data about density-dependence in *Anopheles* is not very comprehensive and therefore it is difficult to draw conclusions about the type of functional response of the population to increasing density. To that end, we tried several functional forms of density-dependence in our model, including exponential, linear, quadratic and log-linear. The exponential form was chosen because it is the best fit to the data and results in mosquito population abundances peaking in the mid-20°C range and larval populations not growing larger than 2000 larvae per liter; these latter two results are more in line with what is known about the biology of these mosquitoes than the results from any of the other functional forms of density-dependence. The exponential form is incorporated in equation 14 (see Text S3, Figures S4, S5, S6, S7, S8, S9, S10, and Tables S1, S2 for results assuming linear form of density-dependence). There is evidence that there may be interactions between temperature and density [45]; however, this relationship has yet to be fully described and therefore can not be incorporated into the model. In our model, we make the simplifying assumption that temperature-dependent mortality is the baseline mortality rate and that the mortality in the larval stage resulting from density-dependence is additive.

The temperature-dependent relationship of the length of parasitic EIP differs from that of the adult mosquito age structure, making it more difficult to predict the temperature at which we would expect to see a peak in mosquitoes that survive to the epidemiologically relevant age. Assuming for simplicity that all mosquitoes become infected as they enter the adult stage, we used both the classic Detinova EIP prediction curve [2], [46] and the curve recently proposed by Paaijmans et al. [7], hereafter in this paper referred to as the Paaijmans curve, to predict the number of mosquitoes that potentially survive to infectiousness. In this study, we are using the EIP predictions based on *P. falciparum* development, because this is the most virulent of the human parasites and the most prevalent in Africa. The Detinova curve was proposed in 1962 and is based on a study of *Plasmodium* development within *An. maculipennis* mosquitoes, a vector of malaria found in Russia. This curve takes the form of a Blunck hyperbola, predicting extremely long development at cool temperatures and fast development at warm temperatures [46]. The Detinova equation is the temperature-dependent parasite development function most used in mechanistic malaria models to date (e.g., [4], [20]). The Paaijmans curve is based on the temperature-development function proposed by Briere et al. [47], which also leads to long development times at cool temperatures but also a slowing and eventual cessation of development beyond the optimum temperature for development. This curve is based on parasite development data from several different *Anopheles* species [7] and takes the upper thermal limit of *P. falciparum* parasites into account. This type of approach has been widely used to explore the effect of temperature on a wide range of ecological and evolutionary questions (e.g., [48]–[50]). These curves differ most substantially at temperatures greater than 26–27°C, which is the warmer end of the parasite development range.

We also compared the predictions our model makes about the potentially infectious age class with predictions made using the “classic” model assumptions of a static, temperature-independent adult mosquito population. For the classic model predictions, we used the same assumptions about the potentially infectious age group as presented above except that to predict survival to that age, we used a constant adult mortality of 10% per day and constant recruitment into the adult stage with no temperature-dependence in either. Additionally, in order to make the predictions comparable, we took the maximum adult recruitment predicted by our model and used that as the recruitment abundance across all temperatures. This assumption means that, given the abundance of adult mosquitoes chosen, the prediction curve will move up or down but the shape will remain the same.

To compare our model predictions with observations of transmission intensity, we compared the number of potentially infectious mosquitoes predicted by our model and those using assumptions of a constant adult mosquito population to observed entomological inoculation rates (EIR) from 14 African countries complied by Mordecai et al. [54]. The EIR is the rate of infectious bites on people and is determined by the following functional relationship:
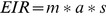
(18)where 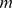 is the number of mosquitoes per host, 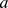 is the daily rate of mosquito biting and 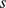 is the proportion of the mosquitoes which have the *Plasmodium* parasites in their salivary glands. Mordecai et al. [54] took EIR observations and matched them with mean transmission-season temperature for each location in the data set, so we can use these to compare our temperature-dependent predictions with estimates of transmission across a range of temperatures.

We ran simulations on the fully parameterized model from 16 to 40°C at one-degree increments, giving 25 different constant temperature runs. This range of temperatures encompasses the temperatures that are relevant for malaria transmission, with 16°C being the lower developmental limit of the malaria parasite *P. falciparum* and 40°C being the thermal death point of mosquitoes [2]. The equilibrium at each temperature was examined to see if the mosquito population was predicted to crash or persist through time. The age structure of the adult population was determined using both the recruitment into the adult stage at equilibrium for each temperature and the adult survival. This was combined with the Detinova and Paaijmans EIP prediction curves to determine the abundance of potentially infectious mosquitoes at each temperature. We ran a local sensitivity analysis looking at the effect of changing each of the 12 parameters on adult recruitment, adult and larval equilibrium abundance and the potentially infectious adult populations using both the Paaijmans and Detinova curves. The sensitivity of the model outputs was calculated using
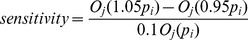
(19)where, 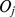 is the model output 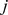 (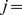 adult, or larval abundance or adult recruitment or potentially infectious adult abundance calculated using the Paaijmans or Detinova curves), and 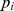 is the parameter 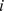 (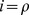, 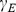, 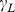, 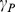, 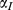, 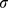, 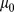, 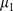, 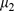, 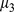, 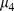, or 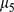). The sensitivity is the percent change in the model output in response to a percentage change in the parameter [51].

## Results

**Figure 2:**
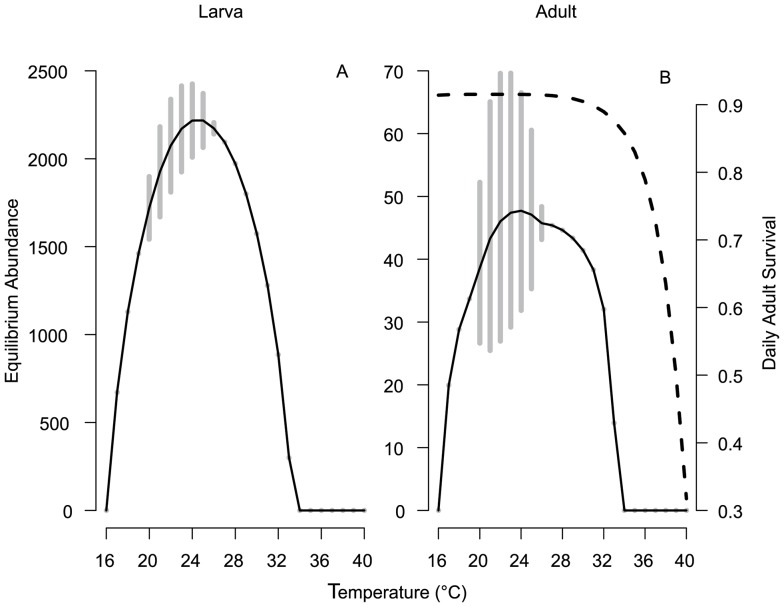
Larval and adult equilibrium abundances. (A) The larval equilibrium abundances across temperatures with exponential density-dependence. (B) The adult equilibrium abundances (solid line, left axis) and daily survival (dashed line, right axis) across temperatures. The gray points and bars in both panels are the stable and cyclic abundances, respectively. The solid line connecting the points is the average abundance across temperature. Notice that the y-axes have different scales.

The model predicts that mosquito populations will persist (i.e. have a population size greater than 1) from 17 to 33°C, which is in line with the experimental data used to parameterize the model [37], [38]. From 17 to 19°C and from 27 to 33°C, mosquito abundance dynamics were predicted to be stable. Between 20 and 26°C, the equilibrium point lost stability and the dynamics followed small amplitude cycles, which are consistent with oscillations seen in other DDE systems [52]. Across the temperature range where populations persisted, the larval equilibrium abundance was 20 to 50 fold the adult abundance (Figure 2). The model predicts that the adult population will be 2.1 to 4.7% the size of the larval population, which is consistent with empirical estimates of larval survival that range between 1 to 10% [23]–[29]. In addition to numerical analysis, we also derived the equilibria analytically. The non-trivial equilibria found by analytical analysis of the model matched the numerical solutions at those temperatures where populations converged on the equilibrium point. Because the model is deterministic with a single attractor, the results are not dependent on the initial conditions.

The cycles displayed by the system from 20 to 26 C result from over-compensatory density-dependence in the larval stage. At these temperatures, the temperature-dependent mortality, which we assume is the baseline mortality, is at its lowest point in both juvenile and adult mosquitoes. Additionally, juvenile development rate is slow to moderate through the range of temperatures in question; this, in combination with low mortality, leads to a large larval population. Density-dependent mortality then becomes very strong and causes the observed over-compensatory crash. The cycles are of such a small amplitude that it is unclear whether it would be discernible in the face of variability in most natural time series of mosquitoes. The cycle period is determined by the length of the juvenile delay (egg to adult maturation time) and corresponds to approximately twice the length of that delay (Table S3). The cycle period is consistent with the dynamics of many insect populations that experience larval competition [52].

Because the adults are the epidemiologically important subset of the mosquito population, we explored the changes in this stage across temperatures and in response to juvenile stage dynamics through the adult recruitment. The juvenile stage mortality rate data showed greater variability to temperature and stayed low over a smaller temperature range than the adult mortality rates. The data suggested that adult mosquito mortality rates did not change much across the temperature range we are interested in, except at the extremely warm temperatures (Figure S2). Daily adult survival is therefore predicted by the model to be high for all temperatures explored except those at the high end of the range. Interestingly, we found adult abundance to be more sensitive to temperature than one would predict based on the adult survivorship alone, having a more defined peak and a sharp decrease in abundance at cooler temperatures (Figure 2). The juvenile stage temperature sensitivities impact recruitment into the adult stage, which made the abundance of adults more temperature-dependent.

**Figure 3:**
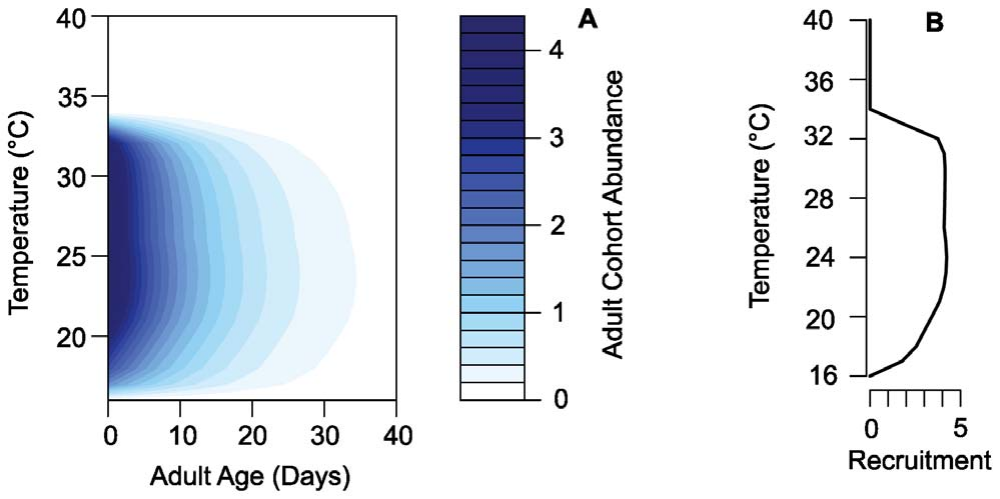
Age-specific adult abundance and adult recruitment across temperature. (A) The age-specific abundance of a single cohort of adult mosquitoes for each temperature. High abundance is in dark blue decreasing to zero in white. (B) Recruitment (the mean abundance of new recruits) into the adult stage over the temperature range.

We explored the effects of temperature and juvenile stage dynamics on adult age structure because it is an important determinant of population vectorial capacity. The combination of temperature-dependencies and intra-stage density-dependence in the larval stage impacted recruitment into the adult stage, and therefore the age structure, in nonlinear ways (Figure 3). The number of mosquitoes emerging as adults was determined by the egg-laying rate and the juvenile stage dynamics. However, because there was no further feedback from the juvenile stages once recruits were in the adult stage, the survival of newly emerged adults was determined by the adult temperature-dependent mortality alone. The model predicted the largest abundance of long-lived mosquitoes to be across the 20–30°C temperature range, with the most noticeable drops in longevity at the extremely warm and cool temperatures where recruitment was low. This corresponds with observations of decreased longevity at temperatures above 32°C and in the East Africa highlands were temperatures are cool [2], [53].

**Figure 4:**
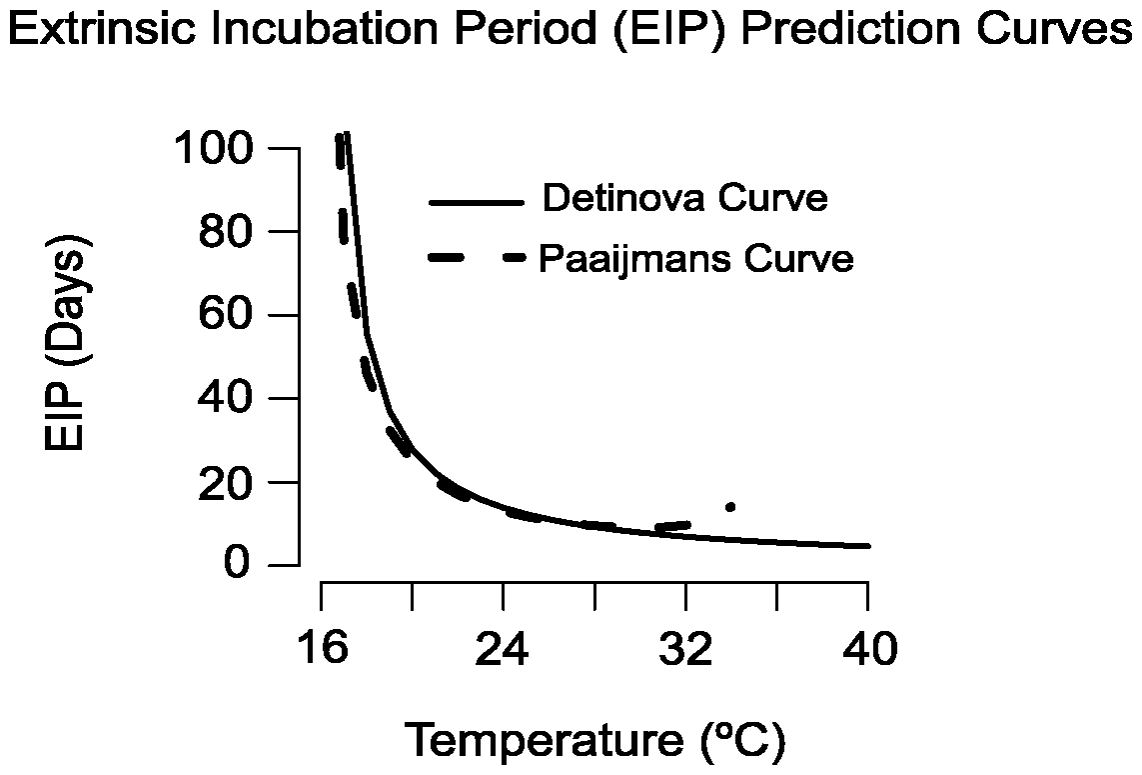
Extrinsic incubation period curves. Temperature-dependent extrinsic incubation period in days; the solid line is the Detinova prediction curve and the dashed line is the Paaijmans curve.

**Figure 5:**
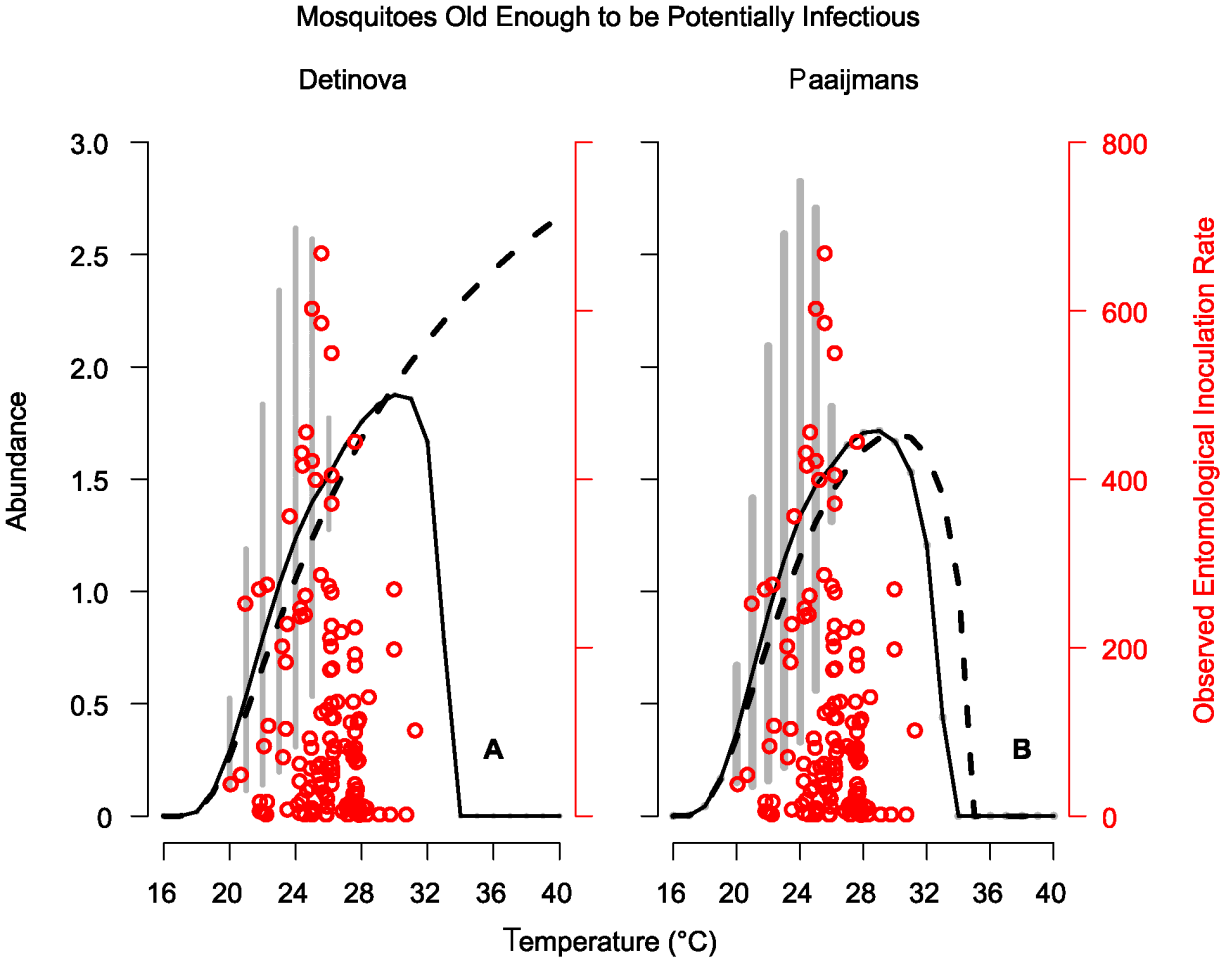
Potential for infectious mosquitoes. The abundance of mosquitoes old enough to be potentially infectious across temperatures. In both graphs the solid line represents the predictions made using our model in combination with either the Detinova (A) or Paaijmans (B) EIP prediction curve. The gray points and bars in both panels are the stable and cyclic abundances predicted by our model, respectively. The dashed lines represent the predictions made using the classic model assumptions of a constant vector population, in combination either the Detinova (A) or Paaijmans (B) EIP prediction curve. The red points, which correspond to the right y-axis are the observed entomological inoculation rates from 14 countries in Africa, compiled by [54].

We found that the adult population old enough to potentially be capable of transmitting malaria was strongly influenced by juvenile stage dynamics through adult recruitment and more strongly temperature-dependent than previously predicted, regardless of the EIP prediction curve used. The predictions about the potentially infectious populations differed based on the EIP prediction curve used, most noticeably at the warmer end of the temperature range. With both the Detinova and the Paaijmans curves (Figure 4), the model predicted a peak in the abundance of mosquitoes potentially able to transmit parasites at cooler temperatures than when using the classic assumptions of a temperature-independent adult population (Figure 5).

The shift to peaks at cooler temperatures is important biologically for this system, because malaria transmission peaks at temperatures in the mid-20s rather than in the 30s [2], [54], [55]. The Detinova curve predicts that parasite development speed continued to increase until it was curtailed by the imposed lethal temperature of 40°C; thus the observed drop in abundance at high temperatures was the result of the population dynamics in the model. Our model, in combination with the Detinova curve, predicted that the abundance of potentially infectious mosquitoes will start to decrease above 30°C. The combination of our model predictions and the Paaijmans curve resulted in a peak in the abundance of epidemiologically relevant mosquitoes at the slightly cooler temperature of 28°C. This observed two-degree drop in the peak occurs because the Paaijmans curve predicts that parasite development will begin to slow above 30°C and eventually halt at 35°C. It should be noted that the Paaijmans curve, because of its predicted 30°C peak, does as well alone in predicting a biologically realistic peak as the Detinova Curve does in combination with our mosquito model. However, the most biologically realistic peak found in this study is the combination of the Paaijmans curve and the predictions of our model. This can be seen when the EIR data points are compared to the predicted results (Figure 5). The transmission intensity is generally higher at cooler temperatures than are predicted by the curves without mosquito dynamics and it drops off rapidly at higher temperatures.

**Figure 6:**
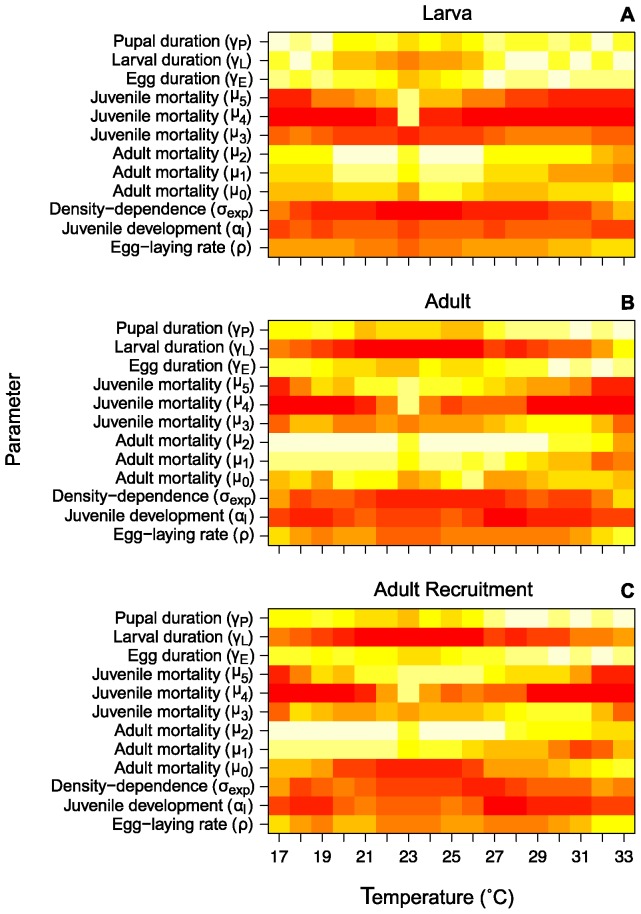
Sensitivity rank across temperature. The sensitivity of larval abundance (A), adult abundance (B) and adult recruitment (C) to changes in the parameters across temperatures ranked from highest to lowest sensitivity. Red indicates greatest sensitivity to change, followed by orange, yellow and white. The x-axis is temperature from 17–33°C, and the y-axis is the parameter.

We ran a local sensitivity analysis on the twelve parameters in our model at each of the 25 temperatures from 16 to 40°C. This allowed us to determine which model parameters were most sensitive to change but also whether the sensitivity of a given parameter changed across the temperature range. The outputs used to gauge model sensitivity to changes in parameters were larval and adult abundance, recruitment into the adult stage and the abundance of mosquitoes old enough to potentially transmit malaria using both the Detinova and Paaijmans EIP prediction curves. For larval abundance the parameters that were the most sensitive to change were the three juvenile mortality parameters (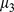, 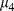, and 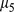), density-dependent mortality (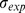) and juvenile development rate (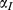). These five parameters displayed high sensitivity across the majority of the temperature range. The parameters that showed the greatest sensitivity for adult abundance were larval density-dependence (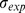), juvenile development rate (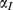) and the proportion of time spent in the larval stage (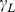). Additionally, adult abundance was sensitive to changes in one of the juvenile mortality parameters (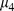), with the other two (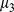, and 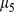) becoming more important at the extreme edges of the temperature range. The responses of adult recruitment and of the potentially infectious mosquito population were quite similar; across most of the temperature range the parameter that resulted in greater sensitivity was the proportion of time spent in the larval stage (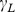). Additionally, across shorter ranges of temperatures juvenile development rate (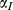), larval density-dependence (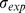), one of the adult mortality parameters (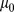), and one of the juvenile parameters (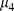), were important (Figure 6, Figures S11, S12, S13, and TablesS4, S5).

## Discussion

The population dynamics and adult age structure of *Anopheles* mosquitoes are important when determining a given population's ability to transmit malaria. Ambient temperature conditions affect both mosquito life history processes and the *Plasmodium* EIP. The sensitivities to temperature change between the mosquito juvenile stages and the adult as well as between life history traits such as development and mortality. Additionally, malaria parasites have a temperature-dependent development curve that does not match up with the mosquito temperature curves. All these factors pull the system in different directions at certain temperatures, making the population response hard to predict. By running simulations of our DDE model across a broad temperature range (16–40°C) we were able to explore population responses to changes in temperature. Our results indicate that non-linear temperature sensitivities throughout the mosquito life cycle have a large impact on the adult population dynamics and therefore on a population's ability to vector malaria effectively. Additionally, our results suggest that juvenile stage dynamics influence adult stage structure dramatically.

Juvenile mosquitoes are not infected by *Plasmodium* parasites and live in an entirely different habitat from the adults, and so juvenile mosquitoes are frequently left out of malaria transmission models. It is well known that conditions experienced by juvenile mosquitoes determine adult characteristics, only some of which have been included in our model [30], [32]–[34]. Our results demonstrate that the effects of temperature on juvenile stages are important in determining the age structure of the adult population. Here we made the simplifying assumption that water and air temperatures are the same. However, it has been been shown that water temperatures in the pools that are preferred by *An. gambiae* mosquitoes are warmer than air temperatures in western Kenya [56]. When data are available across a range of environments, the relationship between air and water temperature can be easily incorporated into the model.

Adult mortality is less sensitive across much of the temperature range in question than the juvenile mortality rate; in fact, across much of the range, mortality in adults is almost constant (Figure S2). If adults were independent of the juvenile stages we would expect to see a very broad flat curve of equilibrium adult abundance. Instead we see a curve that resembles the shape of the larval equilibrium abundance curve and has a more defined peak and rapid declines on both edges of the temperature range (Figure 2). Additionally, our results show that larval density-dependence has a significant regulatory impact on mosquito populations and can lead to low amplitude overcompensation cycles.

Larval density-dependence is an important regulatory process in the model. It is also one of the most sensitive parameters across most of the temperature range for adult recruitment, adult and larval abundance and the abundance of potentially infectious mosquitoes. From the literature, it appears that different species of *Anopheles* respond to increases in density in different ways. For example, *An. stephensi*, an important vector in Southeast Asia, shows an increase in daily mortality rate with increased density in the larval stage [31]. In contrast, *An. gambiae*, an important vector in Africa, does not appear to respond strongly to density through morality rate, but does show a increase in the developmental period [30], [31]. In the model, we assumed that the influence of density is manifested in daily mortality rates and, because of this, used data from *An. stephensi* (Figure S3). This assumption allows for the delays in the model to be determined solely by temperature, making the system more tractable. It also provides a starting point to explore the ways in which the type of density-dependence influences population dynamics.

We also assumed that density-dependence in mortality takes an exponential form. This assumption was based on the fit to the data and on the fact that this form of density-dependence resulted in population abundances and peaks that most closely resemble the biology of the system. Past experimental work has shown a relationship between key life history traits and larval density [30], [31], [33], [34]; however, much of those data are in a format that could not be used to parameterize the model. The data needed to parameterize this model are daily mortality rates for a single *Anopheles* species. The scarcity of data is a great hinderance to understanding *Anopheles* mosquito population dynamics. This is particularly true if we want to use models to predict the potential impact of a mosquito control program, as changing densities of larval populations could have unexpected ramifications in the adult population. The effects of density-dependence on *Anopheles* mosquito populations merit further exploration both theoretically and empirically, as our sensitivity analysis reveals that it is one of the critical parameters.

In addition to density-dependence, other key parameters such as juvenile mortality and development were important for all the population metrics we looked at in our sensitivity analysis. It is interesting to note that all of the parameters that appear to be important to the population across temperatures have been found to be affected by density-dependence [30], [31], [33], [34]. Juvenile mortality and development have been fairly well studied across a constant temperature range in optimized food and density conditions; however, much more data is needed in order to understand how these parameters respond to density and to temperature and density together. The scarcity of field data on density-dependence has recently been addressed by Muriu et al. [57], who found that larval density of *An. gambiae* can impact larval survival and development rates as well as the size of adults. These results provide further evidence that understanding the regulatory processes can increase our understanding of mosquito population dynamics.

The adult population abundance is more sensitive to temperature than previously assumed, such that the adult population only persisted at temperatures that were suitable for juvenile mosquitoes, despite having high predicted survival across a much broader range. This does not correspond directly with the vectorial capacity because the parasitic development rate has a different temperature relationship. We found that the function used to describe *Plasmodium* developmental rate influences the predictions about a mosquito population's ability to effectively transmit the parasite. Both the Detinova and Paaijmans curves predict a rapid increase in developmental rate over the lower end of the temperature range. The Paaijmans curve then tapers off and predicts cessation of development at very high temperatures where the Detinova curve does not. Using either of these curves with the predictions from our model gives a more realistic prediction about temperatures at which we would expect a peak in potentially infectious mosquitoes [2], [54], [55]. Our model predicts that the peak in potentially infectious mosquitoes begins at lower temperatures than models without vector dynamics (Figure 5). This is important for understanding the dynamics of mosquito populations and malaria in the field, and could help to explain some of the mis-match between previous model predictions and observed malaria patterns. The prediction from our model that abundance of potential vectors decreases at high temperatures is a phenomenon that has been demonstrated in malarious regions [2].

One major advantage of having a model such as this one, which admittedly does not include all the aspects of mosquito biology, is that the model is relatively parameter sparse. The inclusion of other types of environmental drivers, mosquito physiological responses, or behavior would add considerably to the degree of difficulty in interpreting the model results. Additionally, many of those relationships are data scarce and we would therefore be in danger of over parameterizing relationships that are not well understood. As it stands, the model represents a starting point and provides a robust framework that can be built upon. Because there are only a dozen parameters in the model, we can point to areas where the sensitivity analysis and/or the data suggest there is good reason for further empirical exploration. The limited number of parameters in our model—and our focus on temperature-dependence—makes it relatively easy to interpret which life history traits are most important for driving dynamics, in contrast to the more parameter heavy models (e.g., [17]).

Despite the availability of control systems, such as drug therapies, insecticides, and bed-nets, malaria continues to be a major problem for a large part of the world. To date, theoretical efforts to understand transmission have in large part failed to take into account vector biology. This is of particular concern when the impacts of climate and/or climate change on disease risk are explored. Our results highlight the importance of including mosquito biology in models of mosquito-borne disease. Incorporating the juvenile stage dynamics increases our understanding of potential for transmission because of strong regulatory effects in the epidemiologically significant adult stage. Furthermore, including temperature-dependencies in the entire life cycle has interesting and non-intuitive impacts on the potential vectorial capacity of a population. The model framework we have developed is robust and can be run with a variety of temperature conditions, including fluctuating temperature regimes (see example in Figure S1). We can also build upon the model, adding processes such as malaria infection. The model is relatively parameter sparse, a considerable bonus for adapting it to different scenarios quickly and effectively. Because all mosquito vectors share the same basic lifecycle, the model can also be converted to other mosquito-borne disease systems, such as Dengue Fever and West Nile Virus. We propose this model as a useful framework to begin to interpret mosquito population responses to temperature sensitivities as well as inter- and intra-stage interactions. Understanding the vector population will lead to clearer understanding of malaria transmission and enhance our ability to predict what may happen to disease intensity in the future.

## Supporting Information

Figure S1 **Adult abundance from model simulations with a 10°C seasonal temperature fluctuation.** (A) Adult abundance trajectory over the course of one year, with a mean temperature of 18°C (B) Adult abundance trajectory over the course of one year, with a mean temperature of 22°C (C) Adult abundance trajectory over the course of one year, with a mean temperature of 26°C (D) Adult abundance trajectory over the course of one year, with a mean temperature of 30°C. The x-axes are all time in days over a single year and the y-axes are adult abundance.
(EPS)

Figure S2 **Developmental and mortality data used in the model parameterization.** (A) Juvenile development rate across temperature. The points are data from [17] and the development function used in our model is the solid line. (B) Development rate of the gonotrophic cycle across temperature. The points are data from [44] and the solid line is the fit function. (C) Temperature-dependent juvenile mortality rate. The filled circles data are from [38] and the x's are data from [37]; the solid line is the fit function. (D) Temperature-dependent adult mortality rate. The filled circles are data from 60% humidity and the x's are data from 80% humidity; these data were published in [36]. The solid line is the fit function used in our model.
(EPS)

Figure S3 **Exponential density-dependence.** Exponential function fit to the larvae mosquito density-dependent daily mortality rate data. The data points are data from *An. stephensi* published in [31].
(EPS)

Figure S4 **Linear density-dependence.** Linear function fit to the larvae mosquito density-dependent daily mortality rate data. The data points are data from *An. stephensi* published in [31].
(EPS)

Figure S5 **Larval and adult equilibrium abundances.** (A) The larval equilibrium abundances across temperatures from the model with linear density-dependence. (B) The adult equilibrium abundances across temperature. Notice that the y-axes have different scales.
(EPS)

Figure S6 **Adult recruitment and age-specific adult abundance across temperature.** (A) The age specific abundance of adult mosquitoes from the model with linear density-dependence. Time in days is on the x-axis, temperature (°C) is on the y-axis. High abundance is in dark blue decreasing to zero in white. (B) The recruitment into the adult stage over the temperature range, with temperature on the y-axis and recruitment on the x-axis.
(TIF)

Figure S7 **Potential for infectious mosquitoes** The abundance of potentially infectious mosquitoes across temperatures from the model with linear density-dependence. In both graphs the solid line represents the predictions made using our model and the dashed line represents the predictions made using the classic model assumptions. The Detinova prediction curve was used to calculate (A), and (B) was calculated using the Paaijmans curve.
(EPS)

Figure S8 **Sensitivity ranks across temperature from the model with linear density-dependence.** The sensitivity of larval abundance (A), adult abundance (B), abundance of potentially infectious mosquitoes using the Detinova (C) and Paaijmans (D) curves, and adult recruitment (E) to changes in the parameters across temperatures ranked from highest to lowest sensitivity. The x-axis is temperature from 17–33°C, and the y-axis is the parameter. Red indicates greatest sensitivity to change, followed by orange, yellow and white.
(EPS)

Figure S9 **Sensitivity analysis of the across temperature from the model with linear density-dependence.** Sensitivity of adult equilibrium abundance, solid black line; larval equilibrium abundance, dashed blue line; and recruitment into the adult stage, dotted green line; across temperature. Temperature from 16–40°C is on the x-axis and sensitivity is on the y-axis.
(EPS)

Figure S10 **Sensitivity analysis of the across temperature from the model with linear density-dependence.** Sensitivity of adult equilibrium abundance, solid black line; larval equilibrium abundance, dashed blue line; and recruitment into the adult stage, dotted green line; across temperature. Temperature from 16–40°C is on the x-axis and sensitivity is on the y-axis.
(EPS)

Figure S11 **Sensitivity rank of the potential for infectious mosquitoes across temperature.** The sensitivity of potentially infectious mosquito population calculated using the Detinova (a) and Paaijmans (b) curves to changes in the parameters across temperatures ranked from highest to lowest sensitivity. The x-axis is temperature from 17–33°C, and the y-axis is the parameter. Red indicates greatest sensitivity to change, followed by orange, yellow and white.
(EPS)

Figure S12 **Sensitivity analysis of the across temperature.** Sensitivity of adult equilibrium abundance, solid black line; larval equilibrium abundance, dashed blue line; and recruitment into the adult stage, dotted green line; across temperature. Temperature from 16–40°C is on the x-axis and sensitivity is on the y-axis.
(EPS)

Figure S13 **Sensitivity analysis of the across temperature.** Sensitivity of adult equilibrium abundance, solid black line; larval equilibrium abundance, dashed blue line; and recruitment into the adult stage, dotted green line; across temperature. Temperature from 16–40°C is on the x-axis and sensitivity is on the y-axis.
(EPS)

Table S1 **Sensitivity values assuming linear density-dependence** The sensitivity values are the percent change in the adult and larval equilibrium abundance and adult recruitment in response to a 5% change in the parameter in the model assuming linear density-dependence.
(PDF)

Table S2 **Potentially infectious abundance sensitivity values assuming linear density-dependence** The sensitivity values are the percent change in the potentially infectious adult abundance calculated using the Detinova or the Paaijmans curve in response to a 5% change in the parameter in the model assuming linear density-dependence.
(PDF)

Table S3 **Periodicity of Fluctuations.**
(PDF)

Table S4 **Sensitivity values assuming exponential density-dependence** The sensitivity values are the percent change in the adult and larval equilibrium abundance and adult recruitment in response to a 5% change in the parameter in the model assuming exponential density-dependence.
(PDF)

Table S5 **Potentially infectious abundance sensitivity values assuming exponential density-dependence** The sensitivity values are the percent change in the potentially infectious adult abundance calculated using the Detinova or the Paaijmans curve in response to a 5% change in the parameter in the model assuming exponential density-dependence.
(PDF)

Text S1 **Model Development and Compression.**
(PDF)

Text S2 **Model Transformation.**
(PDF)

Text S3 **Model results when using the linear form of density-dependence.**
(PDF)

## References

[1] Centers for Disease Control and Prevention (CDC) (2012). Malaria facts. Available: 416 http://www.cdc.gov/malaria/about/facts.html. Accessed 2013 October 11.

[2] Craig MH, Snow RW, le Sueur D (1999) A climate-based distribution model of malaria transmission in sub-Saharan Africa. Parasitol Today 15: 105–111. 10322323 10.1016/s0169-4758(99)01396-4

[3] Gething PW, Smith DL, Patil AP, Tatem AJ, Snow RW, et al (2010) Climate change and the global malaria recession. Nature 465: 342–5. 20485434 10.1038/nature09098 PMC2885436

[4] Guerra CA, Gikandi PW, Tatem AJ, Noor AM, Smith DL, et al (2008) The limits and intensity of *Plasmodium falciparum* transmission: implications for malaria control and elimination worldwide. PLoS Med 5: e38. 18303939 10.1371/journal.pmed.0050038 PMC2253602

[5] Hay SI, Cox J, Rogers DJ, Randolph SE, Stern DI, et al (2002) Climate change and the resurgence of malaria in the East African highlands. Nature 415: 905–9. 11859368 10.1038/415905a PMC3164800

[6] Paaijmans KP, Blanford S, Bell AS, Blanford JI, Read AF, et al (2010) Influence of climate on malaria transmission depends on daily temperature variation. P Natl Acad Sci USA 107: 15135–9. 10.1073/pnas.1006422107 PMC2930540 20696913

[7] Paaijmans KP, Read AF, Thomas MB (2009) Understanding the link between malaria risk and climate. P Natl Acad Sci USA 106: 13844–13849. 10.1073/pnas.0903423106 PMC2720408 19666598

[8] Pascual M, Ahumada JA, Chaves LF, Rodo X, Bouma M (2006) Malaria resurgence in the East African highlands: Temperature trends revisited. P Natl Acad Sci USA 103: 5829–5834. 10.1073/pnas.0508929103 PMC1416896 16571662

[9] Patz JA, Olson SH (2006) Malaria risk and temperature: Influences from global climate change and local land use practices. P Natl Acad Sci USA 103: 5635–5636. 10.1073/pnas.0601493103 PMC1458623 16595623

[10] Rogers DJ, Randolph SE (2000) The global spread of malaria in a future, warmer world. Science 289: 1763–6. 10976072 10.1126/science.289.5485.1763

[11] Ross R (1910) The prevention of malaria. New York: E.P. Dutton and company, 669 pp.

[12] MacDonald G (1957) The Epidemiology and Control of Malaria. Oxford University Press.

[13] Mandal S, Sarkar RR, Sinha S (2011) Mathematical models of malaria - a review. Malaria Journal 10: 202. 21777413 10.1186/1475-2875-10-202 PMC3162588

[14] Smith DL, McKenzie FE (2004) Statics and dynamics of malaria infection in *Anopheles* mosquitoes. Malar J 3: 13. 15180900 10.1186/1475-2875-3-13 PMC449722

[15] Bomblies A, Duchemin JB, Eltahir EAB (2008) Hydrology of malaria: Model development and application to a Sahelian village. Water Resour Res 44.

[16] Bomblies A, Duchemin JB, Eltahir EAB (2009) A mechanistic approach for accurate simulation of village scale malaria transmission. Malar J 8: 223. 19799793 10.1186/1475-2875-8-223 PMC2761400

[17] Depinay JMO, Mbogo CM, Killeen G, Knols B, Beier J, et al (2004) A simulation model of African *Anopheles ecology* and population dynamics for the analysis of malaria transmission. Malar J 3: 29. 15285781 10.1186/1475-2875-3-29 PMC514565

[18] Hancock PA, Godfray HCJ (2007) Application of the lumped age-class technique to studying the dynamics of malaria-mosquito-human interactions. Malar J 6: 98. 17663757 10.1186/1475-2875-6-98 PMC1971713

[19] Hoshen MB, Morse AP (2004) A weather-driven model of malaria transmission. Malar J 3: 32. 15350206 10.1186/1475-2875-3-32 PMC520827

[20] Parham PE, Michael E (2009) Modeling the effects of weather and climate change on malaria transmission. Environ Health Perspect 118. 10.1289/ehp.0901256 PMC2866676 20435552

[21] Beier JC (1998) Malaria parasite development in mosquitoes. Annu Rev En tomol 43: 519–43. 10.1146/annurev.ento.43.1.519 9444756

[22] Boyd MF (1949) Malariology: a comprehensive survey of all aspects of this group of diseases from a global standpoint, volume 1. Philadelphia: Saunders, 1643 pp.

[23] Aniedu I, Mutinga MJ, Mutero CM (1993) Vertical estimates of survivorship of larvae and pupae of *Anopheles gambiae* Giles complex in Baringo District, Kenya. Insect Sci Appl 14: 39–48.

[24] Mukiama TK, Mwangi RW (1989) Field studies of larval *Anopheles arabiensis* Patton of Mwea Irrigation Scheme, Kenya. ICIPE Science Press.

[25] Munga S, Minakawa N, Zhou G, Githeko AK, Yan G (2007) Survivorship of immature stages of *Anopheles gambiae s.l.* (Diptera: Culicidae) in natural habitats in western Kenya highlands. J Med Entomol 44: 758–64. 17915505 10.1603/0022-2585(2007)44[758:soisoa]2.0.co;2

[26] Mwangangi JM, Muturi EJ, Shililu J, Muriu SM, Jacob B, et al (2006) Survival of immature *Anopheles arabiensis* (Diptera: Culicidae) in aquatic habitats in Mwea rice irrigation scheme, central Kenya. Malar J 5: 114. 17125501 10.1186/1475-2875-5-114 PMC1698490

[27] Okogun GRA (2005) Life-table analysis of *Anopheles* malaria vectors: generational mortality as tool in mosquito vector abundance and control studies. J Vector Borne Dis 42: 45–53. 16161700

[28] Service MW (1973) Mortalities of larvae of *Anopheles gambiae* Giles complex and detection of predators by the precipitation test. B Entomol Res 62: 359–369.

[29] Service MW (1977) Mortalities of the immature stages of species B of the *Anopheles gambiae* complex in Kenya: Comparison between rice fields and temporary pools, identification of predators and effects of insecticidal spraying. J Med Entomol 13: 535–545. 845895 10.1093/jmedent/13.4-5.535

[30] Gimnig JE, Ombok M, Otieno S, Kaufman MG, Vulule JM, et al (2002) Density-dependent development of *Anopheles gambiae* (Diptera: Culicidae) larvae in artificial habitats. J Med Entomol 39: 162–72. 11931252 10.1603/0022-2585-39.1.162

[31] Timmermann SE, Briegel H (1993) Water depth and larval density affect development and accumulation of reserves in laboratory populations of mosquitoes. Bull Soc Vector Ecol 18: 174–187.

[32] Grech K, Maung LA, Read AF (2007) The effect of parental rearing conditions on offspring life history in *Anopheles stephensi* . Malar J 6: 130. 17892562 10.1186/1475-2875-6-130 PMC2034587

[33] Lyimo EO, Takken W, Koella JC (1992) Effect of rearing temperature and larval density on larval survival, age at pupation and adult size of *Anopheles gambiae* . Entomol Exp Appl 63: 265–271.

[34] Reisen WK (1975) Intraspecific competition in *Anopheles stephensi* Liston. Mosq News 35: 473–482.

[35] Charlwood JD, Smith T, Billingsley PF, Takken W, Lyimo EOK, et al (1997) Survival and infection probabilities of anthropophagic anophelines from an area of high prevalence of *Plasmodium falciparum* in humans. B Entomol Res 87: 445.

[36] Bayoh MN (2001) Studies on the development and survival of *Anopheles gambiae sensu stricto* at various temperatures and relative humidities. Ph.D. thesis, University of Durham.

[37] Bayoh MN, Lindsay SW (2004) Temperature-related duration of aquatic stages of the Afrotropical malaria vector mosquito *Anopheles gambiae* in the laboratory. Med Vet Entomol 18: 174–9. 15189243 10.1111/j.0269-283X.2004.00495.x

[38] Bayoh MN, Lindsay SW (2003) Effect of temperature on the development of the aquatic stages of *Anopheles gambiae sensu stricto* (Diptera : Culicidae). B Entomol Res 93: 375–381. 10.1079/ber2003259 14641976

[39] Briggs CJ, Godfray HCJ (1995) The dynamics of insect-pathogen interactions in stage-structured populations. Am Nat 145: 855–887.

[40] de Roos AM, Persson L (2003) Competition in size-structured populations: mechanisms inducing cohort formation and population cycles. Theor Popul Biol 63: 1–16. 12464491 10.1016/s0040-5809(02)00009-6

[41] McCauley E, Nelson WA, Nisbet RM (2008) Small-amplitude cycles emerge from stage-structured interactions in daphnia-algal systems. Nature 455: 1240–1243. 18972019 10.1038/nature07220

[42] Nisbet RM, Gurney WSC (1983) The systematic formulation of population-models for insects with dynamically varying instar duration. Theor Popul Biol 23: 114–135.

[43] Yamanaka T, Nelson WA, Uchimura K, Bjornstad ON (2012) Generation separation in simple structured life cycles: Models and 48 years of field data on a tea tortrix moth. Am Nat 179: 95–109. 22173463 10.1086/663201

[44] Lardeux FJ, Tejerina RH, Quispe V, Chavez TK (2008) A physiological time analysis of the duration of the gonotrophic cycle of *Anopheles pseudopunctipennis* and its implications for malaria transmission in Bolivia. Malar J 7: 141. 18655724 10.1186/1475-2875-7-141 PMC2518372

[45] Lyimo EO, Koella JC (1992) Relationship between body size of adult *Anopheles gambiae s.l.* and infection with the malaria parasite *Plasmodium falciparum* . Parasitology 104 (Pt 2) 233–7. 1594289 10.1017/s0031182000061667

[46] Detinova TS (1962) Age-grouping methods in diptera of medical importance with special reference to some vectors of malaria. Monogr Ser World Health Organ 47: 13–191. 13885800

[47] Briere J, Pracros P, Le Roux A, Pierre J (1999) A novel rate model of temperature-dependent development for arthropods. Environmental Entomology 28: 22–29.

[48] Amarasekare P, Savage V (2012) A framework for elucidating the temperature dependence of fitness. Am Nat 179: 178–91. 22218308 10.1086/663677

[49] Frazier MR, Huey RB, Berrigan D (2006) Thermodynamics constrains the evolution of insect population growth rates: “warmer is better”. Am Nat 168: 512–20. 17004222 10.1086/506977

[50] Martin TL, Huey RB (2008) Why “suboptimal” is optimal: Jensen's inequality and ectotherm thermal preferences. Am Nat 171: E102–18. 18271721 10.1086/527502

[51] Ellner SP, Guckenheimer J (2006) Dynamic Models in Biology. Princeton University Press.

[52] Gurney WSC, Nisbet RM (1985) Fluctuation periodicity, generation separation, and the expression of larval competition. Theoretical Population Biology 28: 150–180.

[53] Minakawa N, Omukunda E, Zhou G, Githeko A, Yan G (2006) Malaria vector productivity in relation to the highland environment in Kenya. Am J Trop Med Hyg 75: 448–453. 16968920

[54] Mordecai EA, Paaijmans KP, Johnson LR, Balzer C, Ben-Horin T, et al (2012) Optimal temperature for malaria transmission is dramatically lower than previously predicted. Ecology Letters 16: 22–30. 23050931 10.1111/ele.12015

[55] Paaijmans KP, Blanford S, Chan BHK, Thomas MB (2012) Warmer temperatures reduce the vectorial capacity of malaria mosquitoes. Biology letters 8: 465–468. 22188673 10.1098/rsbl.2011.1075 PMC3367745

[56] Paaijmans KP, Jacobs AFG, Takken W, Heusinkveld BG, Githeko AK, et al (2008) Observations and model estimates of diurnal water temperature dynamics in mosquito breeding sites in western Kenya. Hydrological Processes 22: 4789–4801.

[57] Muriu SM, Coulson T, Mbogo CM, Godfray HCJ (2012) Larval density dependence in *Anopheles gambiae* s.s., the major African vector of malaria. Journal of Animal Ecology 82: 166–174. 23163565 10.1111/1365-2656.12002 PMC5373432

[58] Beaty BJ, Marquardt WC (1996) The biology of disease vectors. Niwot, Colo.: University Press of Colorado.

[59] Clements AN (1992) The biology of mosquitoes. London: Chapman & Hall, 1st ed edition.

[60] Lyimo EO, Takken W (1993) Effects of adult body size on fecundity and the pre-gravid rate of *Anopheles gambiae* females in Tanzania. Med Vet Entomol 7: 328–332. 8268486 10.1111/j.1365-2915.1993.tb00700.x

