# Supplementary figures and images for: The Effect of Temperature on Anopheles Mosquito Population Dynamics and the Potential for Malaria Transmission
Source: PLoS One. 2013 Nov 14;8(11):e79276. doi: 10.1371/journal.pone.0079276 (PMC3828393; doi:10.1371/journal.pone.0079276)

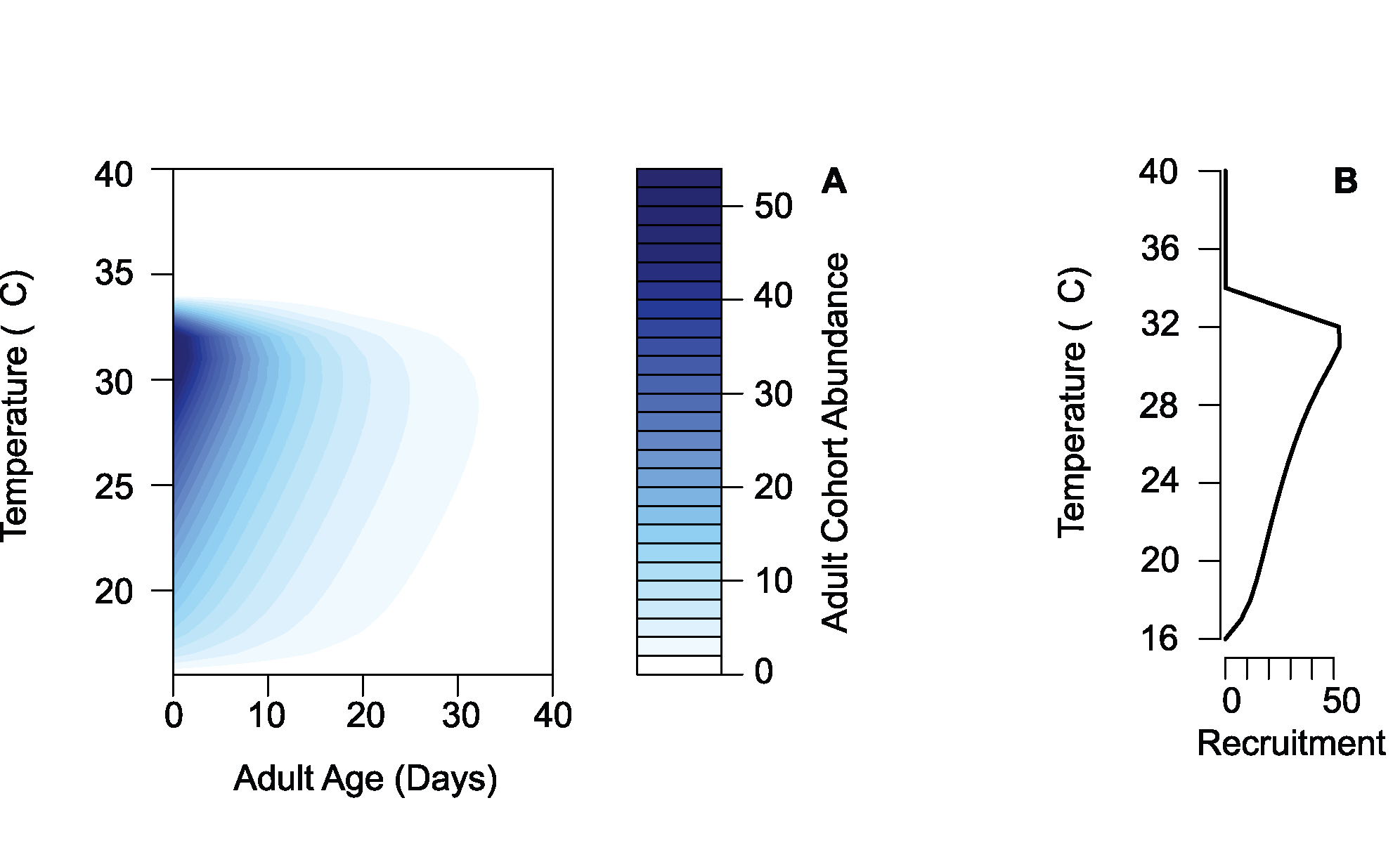

Supplement: Figure S6 — Adult recruitment and age-specific adult abundance across temperature. (A) The age specific abundance of adult mosquitoes from the model with linear density-dependence. Time in days is on the x-axis, temperature (°C) is on the y-axis. High abundance is in dark blue decreasing to zero in white. (B) The recruitment into the adult stage over the temperature range, with temperature on the y-axis and recruitment on the x-axis. (TIF) [file pone.0079276.s006.tif]
