# Supplementary material for: The Effect of Temperature on Anopheles Mosquito Population Dynamics and the Potential for Malaria Transmission
Source: PLoS One. 2013 Nov 14;8(11):e79276. doi: 10.1371/journal.pone.0079276 (PMC3828393; doi:10.1371/journal.pone.0079276)
Supplement: Table S1 — Sensitivity values assuming linear density-dependence The sensitivity values are the percent change in the adult and larval equilibrium abundance and adult recruitment in response to a 5% change in the parameter in the model assuming linear density-dependence. (PDF) [file pone.0079276.s014.pdf]

Table S1. Sensitivity values assuming linear density-dependence The sensitivity values are the percent change in the adult and larval equilibrium abundance and adult recruitment in response to a 5% change in the parameter in the model assuming linear density-dependence.

Parameter	20°C	24°C	28°C	32°C
**Adult Sensitivity Values**				
ρ	$-5.65\text{e}-1$	$-6.28\text{e}-1$	$-6.25\text{e}-1$	$-3.90\text{e}-1$
α_I	2.05	1.98	1.98	2.71
σ_{lin}	-1.00	-1.00	-1.00	-1.00
μ_0	$-4.36\text{e}-1$	$-3.37\text{e}-1$	$-3.76\text{e}-1$	$-6.11\text{e}-1$
μ_1	$-2.38\text{e}-3$	$1.61\text{e}-2$	$1.09\text{e}-1$	1.34
μ_2	$7.80\text{e}-5$	$1.87\text{e}-3$	$6.63\text{e}-2$	$6.85\text{e}-1$
μ_3	$-5.21\text{e}-2$	$1.80\text{e}-2$	$2.07\text{e}-2$	$-7.10\text{e}-2$
μ_4	$-2.01\text{e}-1$	$-1.88\text{e}-2$	$-8.46\text{e}-2$	10.44
μ_5	$2.24\text{e}-2$	$-8.58\text{e}-4$	$-2.41\text{e}-2$	3.02
γ_E	$2.50\text{e}-1$	$2.50\text{e}-1$	$2.50\text{e}-1$	$2.50\text{e}-1$
γ_L	-2.01	-2.01	-2.01	-2.01
γ_P	$2.50\text{e}-1$	$2.50\text{e}-1$	$2.50\text{e}-1$	$2.50\text{e}-1$
**Larval Sensitivity Values**				
ρ	$2.35\text{e}-1$	$1.94\text{e}-1$	$1.97\text{e}-1$	$3.73\text{e}-1$
α_I	1.34	1.17	1.24	2.34
σ_{lin}	-1.00	-1.00	-1.00	-1.00
μ_0	$-2.35\text{e}-1$	$-1.94\text{e}-1$	$-1.97\text{e}-1$	$-3.73\text{e}-1$
μ_1	$-1.28\text{e}-3$	$8.37\text{e}-3$	$1.10\text{e}-1$	$8.23\text{e}-1$
μ_2	$4.20\text{e}-5$	$9.74\text{e}-4$	$3.48\text{e}-2$	$4.20\text{e}-1$
μ_3	$-3.44\text{e}-1$	$-1.72\text{e}-1$	$-2.37\text{e}-1$	$-1.34\text{e}-1$
μ_4	-1.17	$-1.93\text{e}-1$	1.35	14.09
μ_5	$1.48\text{e}-1$	$8.18\text{e}-3$	$2.83\text{e}-1$	5.27
γ_E	$1.25\text{e}-1$	$1.25\text{e}-1$	$1.25\text{e}-1$	$1.25\text{e}-1$
γ_L	-1.00	-1.00	-1.00	-1.00
γ_P	$1.25\text{e}-1$	$1.25\text{e}-1$	$1.25\text{e}-1$	$1.25\text{e}-1$
**Adult Recruitment Sensitivity Values**				
ρ	$-5.65\text{e}-1$	$-6.28\text{e}-1$	$-6.25\text{e}-1$	$-3.90\text{e}-1$
α_I	2.05	1.98	1.98	2.71
σ_{lin}	-1.00	-1.00	-1.00	-1.00
μ_0	$5.64\text{e}-1$	$6.28\text{e}-1$	$6.25\text{e}-1$	$3.90\text{e}-1$
μ_1	$3.08\text{e}-3$	$-2.71\text{e}-2$	$-3.50\text{e}-1$	$-8.59\text{e}-1$
μ_2	$-1.01\text{e}-4$	$-3.15\text{e}-3$	$-1.10\text{e}-1$	$-4.39\text{e}-1$
μ_3	$-5.21\text{e}-2$	$1.80\text{e}-2$	$2.07\text{e}-2$	$-7.10\text{e}-1$
μ_4	$-2.01\text{e}-1$	$1.88\text{e}-2$	$-8.46\text{e}-2$	10.44
μ_5	$2.24\text{e}-2$	$-8.58\text{e}-4$	$-2.41\text{e}-2$	3.02
γ_E	$2.50\text{e}-1$	$2.50\text{e}-1$	$2.50\text{e}-1$	$2.50\text{e}-1$
γ_L	-2.01	-2.01	-2.01	-2.01
γ_P	$2.50\text{e}-1$	$2.50\text{e}-1$	$2.50\text{e}-1$	$2.50\text{e}-1$
