# Supplementary material for: The Effect of Temperature on Anopheles Mosquito Population Dynamics and the Potential for Malaria Transmission
Source: PLoS One. 2013 Nov 14;8(11):e79276. doi: 10.1371/journal.pone.0079276 (PMC3828393; doi:10.1371/journal.pone.0079276)
Supplement: Table S2 — Potentially infectious abundance sensitivity values assuming linear density-dependence The sensitivity values are the percent change in the potentially infectious adult abundance calculated using the Detinova or the Paaijmans curve in response to a 5% change in the parameter in the model assuming linear density-dependence. (PDF) [file pone.0079276.s015.pdf]

Table S2. Potentially infectious abundance sensitivity values assuming linear density-dependence The sensitivity values are the percent change in the potentially infectious adult abundance calculated using the Detinova or the Paaijmans curve in response to a 5% change in the parameter in the model assuming linear density-dependence.

Parameter	20°C	24°C	28°C	32°C
**Sensitivity Values using Detinova Curve**				
ρ	$-5.65e-1$	$-6.28e-1$	$-6.25e-1$	$-3.90e-1$
α_I	2.05	1.98	1.98	2.71
σ_{lin}	-1.00	-1.00	-1.00	-1.00
μ_0	-1.90	$-6.02e-1$	$-2.31e-1$	$-4.21e-1$
μ_1	$-1.04e-2$	$2.62e-2$	$1.33e-1$	$9.62e-1$
μ_2	$3.40e-4$	$3.04e-3$	$4.13e-2$	$4.83e-1$
μ_3	$-5.21e-2$	$-1.89e-2$	$2.07e-2$	$-7.10e-1$
μ_4	$-2.01e-1$	$1.88e-2$	$-8.46e-2$	10.44
μ_5	$2.24e-2$	$-8.58e-4$	$-2.41e-2$	3.02
γ_E	$2.50e-1$	$2.50e-1$	$2.50e-1$	$2.50e-1$
γ_L	-2.01	-2.01	-2.01	-2.01
γ_P	$2.50e-1$	$2.50e-1$	$2.50e-1$	$2.50e-1$
**Sensitivity Values using Paaijmans Curve**				
ρ	$-5.65e-1$	$-6.28e-1$	$-6.25e-1$	$-3.90e-1$
α_I	2.05	1.98	1.98	2.71
σ_{lin}	-1.00	-1.00	-1.00	-1.00
μ_0	-1.65	$-5.26e-1$	$-2.59e-1$	$-7.44e-1$
μ_1	$-9.03e-3$	$2.29e-2$	$1.49e-1$	1.67
μ_2	$2.96e-4$	$2.66e-3$	$4.63e-2$	$8.46e-1$
μ_3	$-5.21e-2$	$-1.89e-2$	$2.07e-2$	$-7.10e-1$
μ_4	$-2.01e-1$	$1.88e-2$	$-8.46e-2$	10.44
μ_5	$2.24e-2$	$-8.58e-4$	$-2.41e-2$	3.02
γ_E	$2.50e-1$	$2.50e-1$	$2.50e-1$	$2.50e-1$
γ_L	-2.01	-2.01	-2.01	-2.01
γ_P	$2.50e-1$	$2.50e-1$	$2.50e-1$	$2.50e-1$
