# Supplementary material for: The Effect of Temperature on Anopheles Mosquito Population Dynamics and the Potential for Malaria Transmission
Source: PLoS One. 2013 Nov 14;8(11):e79276. doi: 10.1371/journal.pone.0079276 (PMC3828393; doi:10.1371/journal.pone.0079276)
Supplement: Table S3 — Periodicity of Fluctuations. (PDF) [file pone.0079276.s016.pdf]

Table S3. Periodicity of Fluctuations

Temperature	Generation Time (Days)	Period (Days)	2 x Juvenile Development Delay
20	31.08	36.93	39.59
21	29.48	34.98	36.39
22	28.08	32.78	33.58
23	26.84	30.80	31.10
24	25.72	28.61	28.90
25	24.71	27.06	26.93
26	23.75	25.29	25.17
