# Supplementary material for: The Effect of Temperature on Anopheles Mosquito Population Dynamics and the Potential for Malaria Transmission
Source: PLoS One. 2013 Nov 14;8(11):e79276. doi: 10.1371/journal.pone.0079276 (PMC3828393; doi:10.1371/journal.pone.0079276)
Supplement: Table S4 — Sensitivity values assuming exponential density-dependence The sensitivity values are the percent change in the adult and larval equilibrium abundance and adult recruitment in response to a 5% change in the parameter in the model assuming exponential density-dependence. (PDF) [file pone.0079276.s017.pdf]

Table S4. Sensitivity values assuming exponential density-dependence The sensitivity values are the percent change in the adult and larval equilibrium abundance and adult recruitment in response to a 5% change in the parameter in the model assuming exponential density-dependence.

**Parameter**	**20°C**	**24°C**	**28°C**	**32°C**
**Adult Sensitivity Values**				
ρ	$-4.06e-1$	$-5.64e-1$	$-7.53e-1$	$-5.44e-1$
α_I	1.02	$7.60e-1$	1.18	1.75
σ_{exp}	-1.00	-1.00	-1.00	-1.00
μ_0	$9.88e-2$	$1.90e-1$	$-2.49e-1$	$4.57e-1$
μ_1	$-2.19e-3$	$-8.46e-3$	$1.39e-1$	1.00
μ_2	$9.68e-7$	$-9.83e-4$	$4.38e-2$	$5.13e-1$
μ_3	$-7.27e-1$	$-3.93e-1$	$-1.81e-1$	$-7.46e-1$
μ_4	$-1.77e-1$	$4.29e-1$	$9.93e-1$	10.91
μ_5	$3.11e-1$	$1.80e-2$	$2.15e-1$	$3.13e-1$
γ_E	$2.11e-1$	$1.89e-1$	$1.25e-1$	$1.25e-1$
γ_L	-1.64	-1.59	-1.00	-1.00
γ_P	$2.11e-1$	$1.89e-1$	$1.25e-1$	$1.25e-1$
**Larval Sensitivity Values**				
ρ	$1.29e-1$	$9.04e-1$	$6.99e-2$	$2.20e-1$
α_I	$5.11e-1$	$3.23e-1$	$4.39e-1$	1.38
σ_{exp}	-1.00	-1.00	-1.00	-1.00
μ_0	$-5.10e-2$	$-9.09e-3$	$-6.99e-2$	$-2.20e-1$
μ_1	$9.63e-5$	$3.37e-4$	$3.92e-2$	$4.86e-1$
μ_2	$-2.56e-5$	$1.33e-5$	$1.23e-2$	$2.48e-1$
μ_3	$-5.88e-1$	$-4.05e-1$	$-4.39e-1$	-1.39
μ_4	-1.85	$4.44e-1$	2.40	14.74
μ_5	$2.50e-1$	$1.92e-3$	$5.22e-1$	$5.40e-1$
γ_E	$1.06e-2$	$1.02e-2$	$-1.84e-10$	$-4.44e-10$
γ_L	$-7.22e-2$	$-8.10e-2$	$5.20e-12$	$-4.69e-10$
γ_P	$1.07e-2$	$1.02e-2$	$-1.86e-10$	$-4.13e-10$
**Adult Recruitment Sensitivity Values**				
ρ	$-4.24e-1$	$-5.55e-1$	$-7.53e-1$	$-5.44e-1$
α_I	1.01	$7.72e-1$	1.18	1.75
σ_{exp}	-1.00	-1.00	-1.00	-1.00
μ_0	1.09	1.20	$7.52e-1$	$5.44e-1$
μ_1	$-1.05e-3$	$-3.57e-2$	$-4.21e-1$	-1.20
μ_2	$6.04e-5$	$-5.58e-3$	$-1.33e-1$	$-6.13e-1$
μ_3	$-7.21e-1$	$-4.20e-1$	$-1.81e-1$	$-7.46e-1$
μ_4	-1.78	$-4.38e-1$	$9.93e-1$	10.91
μ_5	$3.24e-1$	$2.03e-2$	$2.15e-1$	3.13
γ_E	$2.10e-1$	$1.64e-1$	$1.25e-1$	$1.25e-1$
γ_L	-1.62	-1.60	-1.00	-1.00
γ_P	$2.10e-1$	$1.64e-1$	$1.25e-1$	$1.25e-1$
