# Supplementary material for: The Effect of Temperature on Anopheles Mosquito Population Dynamics and the Potential for Malaria Transmission
Source: PLoS One. 2013 Nov 14;8(11):e79276. doi: 10.1371/journal.pone.0079276 (PMC3828393; doi:10.1371/journal.pone.0079276)
Supplement: Table S5 — Potentially infectious abundance sensitivity values assuming exponential density-dependence The sensitivity values are the percent change in the potentially infectious adult abundance calculated using the Detinova or the Paaijmans curve in response to a 5% change in the parameter in the model assuming exponential density-dependence. (PDF) [file pone.0079276.s018.pdf]

Table S5. Potentially infectious abundance sensitivity values assuming exponential density-dependence The sensitivity values are the percent change in the potentially infectious adult abundance calculated using the Detinova or the Paaijmans curve in response to a 5% change in the parameter in the model assuming exponential density-dependence.

Parameter	20°C	24°C	28°C	32°C
**Sensitivity Values using Detinova Curve**				
ρ	$-4.24e-1$	$-5.55e-1$	$-7.53e-1$	$-5.44e-1$
α_I	1.01	$7.72e-1$	1.18	1.75
σ_{exp}	-1.00	-1.00	-1.00	-1.00
μ_0	1.09	1.20	$7.52e-1$	$5.44e-1$
μ_1	$-1.05e-3$	$-3.57e-2$	$-4.21e-1$	-1.20
μ_2	$6.04e-5$	$-5.58e-3$	$-1.33e-1$	$-6.13e-1$
μ_3	$-7.21e-1$	$-4.20e-1$	$-1.81e-1$	$-7.46e-1$
μ_4	-1.78	$-4.38e-1$	$9.93e-1$	10.91
μ_5	$3.24e-1$	$2.03e-2$	$2.15e-1$	3.13
γ_E	$2.10e-1$	$1.64e-1$	$1.25e-1$	$1.25e-1$
γ_L	-1.62	-1.60	-1.00	-1.00
γ_P	$2.10e-1$	$1.64e-1$	$1.25e-1$	$1.25e-1$
**Sensitivity Values using Paaijmans Curve**				
ρ	$-4.24e-1$	$-5.55e-1$	$-7.53e-1$	$-5.44e-1$
α_I	1.01	$7.72e-1$	1.18	1.75
σ_{exp}	-1.00	-1.00	-1.00	-1.00
μ_0	1.09	1.20	$7.52e-1$	$5.44e-1$
μ_1	$-1.05e-3$	$-3.57e-2$	$-4.21e-1$	-1.20
μ_2	$6.04e-5$	$-5.58e-3$	$-1.33e-1$	$-6.13e-1$
μ_3	$-7.21e-1$	$-4.20e-1$	$-1.81e-1$	$-7.46e-1$
μ_4	-1.78	$-4.38e-1$	$9.93e-1$	10.91
μ_5	$3.24e-1$	$2.03e-2$	$2.15e-1$	3.13
γ_E	$2.10e-1$	$1.64e-1$	$1.25e-1$	$1.25e-1$
γ_L	-1.62	-1.60	-1.00	-1.00
γ_P	$2.10e-1$	$1.64e-1$	$1.25e-1$	$1.25e-1$
