# Supplementary material for: The Effect of Temperature on Anopheles Mosquito Population Dynamics and the Potential for Malaria Transmission
Source: PLoS One. 2013 Nov 14;8(11):e79276. doi: 10.1371/journal.pone.0079276 (PMC3828393; doi:10.1371/journal.pone.0079276)
Supplement: Text S1 — Model Development and Compression. (PDF) [file pone.0079276.s019.pdf]

Text S1. Model Development and Compression

The full mosquito population model includes four state equations that follow the four main stages of the lifecycle: egg, larva, pupa and adult. In this form, however, the model is quite large and cumbersome. We can make the model easier to work with by compressing the four state equations into two. By choosing the larval and adult stages to follow, we can continue to track the density-dependence in the larval stage and the epidemiologically important adult stage. We retain the information from the egg and pupal stages by incorporating the information into the larval and adult state equations through nested delays. The equations can be expanded at any time.

The full population model is as follows:

$$\frac{dE(t)}{dt} = R_E(t) - R_L(t) - \delta_E(t)E(t) \tag{S1}$$

$$\frac{dL(t)}{dt} = R_L(t) - R_P(t) - \delta_L(t)L(t) \tag{S2}$$

$$\frac{dP(t)}{dt} = R_P(t) - R_A(t) - \delta_P(t)P(t) \tag{S3}$$

$$\frac{dA(t)}{dt} = R_A(t) - \delta_A(t)A(t) \tag{S4}$$

where,

$$\begin{aligned}
R_E(t) &= b(t)A(t) \\
R_L(t) &= R_E(t-\tau_E(t))S_E(t)\frac{h_E(t)}{h_E\left(t-\tau_E(t)\right)} \\
R_P(t) &= R_L(t-\tau_L(t))S_L(t)\frac{h_L(t)}{h_L\left(t-\tau_L(t)\right)} \\
R_A(t) &= R_P(t-\tau_P(t))S_P(t)\frac{h_P(t)}{h_P\left(t-\tau_P(t)\right)}
\end{aligned}$$

The stage durations are given by:

$$
\begin{aligned}
1 &= \int_{t-\tau_E(t)}^{t} h_E(\xi)d\xi \\
1 &= \int_{t-\tau_L(t)}^{t} h_L(\xi)d\xi \\
1 &= \int_{t-\tau_P(t)}^{t} h_P(\xi)d\xi \\
1 &= \int_{t-\tau_A(t)}^{t} h_A(\xi)d\xi
\end{aligned}
$$

Currently the stage durations are all set such that the integral over the delay is equal to one. Because of the way the data are set up with egg to adult development [17] , it is sensible make the integral over the entire juvenile period equal to one.

$$
\begin{aligned}
h_I(t) &= \alpha_I T(t)^{\beta} \\
1 &= \int_{t-\tau_I(t)}^{t} h_I(\xi)d\xi
\end{aligned}
$$

where $h_I(t)$ is the development rate from egg to adult emergence. In order to break up the development into the three juvenile stages we assume that:

$$
\begin{aligned}
h_E(t) &= \gamma_E h_I(t) \\
h_L(t) &= \gamma_L h_I(t) \\
h_P(t) &= \gamma_P h_I(t)
\end{aligned}
$$

where,

$$
1 = \frac{1}{\gamma_E} + \frac{1}{\gamma_L} + \frac{1}{\gamma_P}
$$

therefore,

$$
\begin{aligned}
\frac{1}{\gamma_E} &= \int_{t-\tau_E(t)}^{t} h_I(\xi) d\xi \\
\frac{1}{\gamma_L} &= \int_{t-\tau_L(t)}^{t} h_I(\xi) d\xi \\
\frac{1}{\gamma_P} &= \int_{t-\tau_P(t)}^{t} h_I(\xi) d\xi
\end{aligned}
$$

so,

$$
\begin{aligned}
\frac{1}{\gamma_E} &= \int_{t-\tau_E(t)}^{t} \alpha_I T(\xi)^{\beta} d\xi \\
\frac{1}{\gamma_L} &= \int_{t-\tau_L(t)}^{t} \alpha_I T(\xi)^{\beta} d\xi \\
\frac{1}{\gamma_P} &= \int_{t-\tau_P(t)}^{t} \alpha_I T(\xi)^{\beta} d\xi
\end{aligned}
$$

where, γ_i is the proportion of the total juvenile development time that stage i makes up.

If we assume a power function describes the development for the juvenile stages and the gonotrophic cycle we get,

$$
\begin{aligned}
R_E(t) &= b(t)A(t) \\
R_L(t) &= R_E(t-\tau_E(t))S_E(t)\frac{T(t)^{\beta}}{T\left(t-\tau_E(t)\right)^{\beta}} \\
R_P(t) &= R_L(t-\tau_L(t))S_L(t)\frac{T(t)^{\beta}}{T\left(t-\tau_L(t)\right)^{\beta}} \\
R_A(t) &= R_P(t-\tau_P(t))S_P(t)\frac{T(t)^{\beta}}{T\left(t-\tau_P(t)\right)^{\beta}}
\end{aligned}
$$

Recall,the stage survivorships are given by

$$
\begin{aligned}
S_E(t) &= \exp\left(-\int_{t-\tau_E(t)}^{t} \delta_E(\xi)d\xi\right) \\
S_L(t) &= \exp\left(-\int_{t-\tau_L(t)}^{t} \delta_L(\xi)d\xi\right) \\
S_P(t) &= \exp\left(-\int_{t-\tau_P(t)}^{t} \delta_P(\xi)d\xi\right) \\
S_A(t) &= \exp\left(-\int_{t-\tau_A(t)}^{t} \delta_A(\xi)d\xi\right)
\end{aligned}
$$

So, putting the recruitment equations together we get:

$$
\begin{aligned}
R_E(t) &= b(t)A(t) \\
R_L(t) &= b(t-\tau_E(t))A(t-\tau_E(t))S_E(t)\frac{T(t)^\beta}{T\left(t-\tau_E(t)\right)^\beta} \\
R_P(t) &= b(t-\tau_L(t)-\tau_E(t-\tau_L(t)))A(t-\tau_L(t)-\tau_E(t-\tau_L(t))) \\
&\quad S_E(t-\tau_L(t))\frac{T(t-\tau_L(t))^\beta}{T\left(t-\tau_L(t)-\tau_E(t-\tau_L(t))\right)^\beta} \\
&\quad S_L(t)\frac{T(t)^\beta}{T\left(t-\tau_L(t)\right)^\beta} \\
R_A(t) &= b(t-\tau_P(t)-\tau_L(t-\tau_P(t))-\tau_E(t-\tau_P(t)-\tau_L(t-\tau_P(t)))) \\
&\quad A(t-\tau_P(t)-\tau_L(t-\tau_P(t))-\tau_E(t-\tau_P(t)-\tau_L(t-\tau_P(t)))) \\
&\quad S_E(t-\tau_P(t)-\tau_L(t-\tau_P(t)))\frac{T(t-\tau_P(t)-\tau_L(t-\tau_P(t)))^\beta}{T\left(t-\tau_P(t)-\tau_L(t-\tau_P(t))-\tau_E(t-\tau_P(t)-\tau_L(t-\tau_P(t)))\right)^\beta} \\
&\quad S_L(t-\tau_P(t))\frac{T(t-\tau_P(t))^\beta}{T\left(t-\tau_P(t)-\tau_L(t-\tau_P(t))\right)^\beta}S_P(t)\frac{T(t)^\beta}{T\left(t-\tau_P(t)\right)^\beta}
\end{aligned}
$$

It is now helpful to define the cumulative delays:

$$
\begin{aligned}
w_1 &= t - \tau_E(t) \\
w_2 &= t - \tau_L(t) \\
w_3 &= t - \tau_P(t) \\
w_4 &= t - \tau_L(t) - \tau_E(t - \tau_L(t)) \\
w_5 &= t - \tau_P(t) - \tau_L(t - \tau_P(t)) \\
w_6 &= t - \tau_P(t) - \tau_L(t - \tau_P(t)) - \tau_E(t - \tau_P(t) - \tau_L(t - \tau_P(t)))
\end{aligned}
$$

where, w_i is the cumulative delay. We can also combine the survivorship functions as follows:

$$
\begin{aligned}
S_1(t) &= \exp\left(-\int_{w_1}^{t} \delta_E(\xi)d\xi\right) \\
S_2(t) &= \exp\left(-\int_{w_4}^{w_2} \delta_E(\xi)d\xi - \int_{w_2}^{t} \delta_L(\xi)d\xi\right) \\
S_3(t) &= \exp\left(-\int_{w_6}^{w_5} \delta_E(\xi)d\xi - \int_{w_5}^{w_3} \delta_L(\xi)d\xi - \int_{w_3}^{t} \delta_P(\xi)d\xi\right)
\end{aligned}
$$

Putting these in to the above recruitment equations and by canceling out some of the development functions we get:

$$
\begin{aligned}
R_E(t) &= b(t)A(t) \\
R_L(t) &= b(w_1)A(w_1)S_1(t)\frac{T(t)^{\beta}}{T\left(w_1\right)^{\beta}} \\
R_P(t) &= b(w_4)A(w_4)S_2(t)\frac{T(t)^{\beta}}{T\left(w_4\right)^{\beta}} \\
R_A(t) &= b(w_6)A(w_6)S_3(t)\frac{T(t)^{\beta}}{T\left(w_6\right)^{\beta}}
\end{aligned}
$$

Now we can compress the model from four state equations to two state equations following the larval and adult stages. This is only possible because of the nested delays, which allow us, in essence, to look

into the past juvenile stages without actually following the state equation. So going back to equations $S1 - S4$ and putting in the simplified recruitment equations gives:

$$\frac{dL(t)}{dt} = b(w_1)A(w_1)S_1(t)\frac{T(t)^\beta}{T\left(w_1\right)^\beta} - b(w_4)A(w_4)S_2(t)\frac{T(t)^\beta}{T\left(w_4\right)^\beta} - \delta_L(t)L(t) \tag{S5}$$

$$\frac{dA(t)}{dt} = b(w_6)A(w_6)S_3(t)\frac{T(t)^\beta}{T\left(w_6\right)^\beta} - \delta_A(t)A(t) \tag{S6}$$
