# Supplementary material for: The Effect of Temperature on Anopheles Mosquito Population Dynamics and the Potential for Malaria Transmission
Source: PLoS One. 2013 Nov 14;8(11):e79276. doi: 10.1371/journal.pone.0079276 (PMC3828393; doi:10.1371/journal.pone.0079276)
Supplement: Text S2 — Model Transformation. (PDF) [file pone.0079276.s020.pdf]

Text S2. Model Transformation

The delays in the mosquito population model are dependent on temperature, as are the mortality and egg-laying rates. Because of the dependence on temperature, the delays change from fixed delays to variable delays when temperature changes through time. Variable delays will make the model much more difficult to work with. To address this problem, we can transform the model onto the physiological time scale, ϕ. When we run the model on this scale most of the temperature-dependence drops out and it becomes a fixed delay model again [41, 43]. The transformation is as follows: let,

$$\phi(t) = \int_0^t T(\xi)^\beta d\xi \tag{S7}$$

$$m(\phi(t)) = T(t)^\beta \tag{S8}$$

Now differentiate equation $S7$ with respect to t:

$$\frac{d\phi}{dt} = T(t)^\beta$$

Putting in equation $S8$ we get,

$$\frac{d\phi}{dt} = m(\phi)$$

Rearrange to get,

$$\frac{dt}{d\phi} = \frac{1}{m(\phi)} \tag{S9}$$

Now we can re-write the stage durations

$$
\begin{aligned}
\phi(t-\tau_j(t)) &= \int_0^{t-\tau_j(t)} T(\xi)^\beta d\xi \\
&= \int_0^{t} T(\xi)^\beta d\xi - \int_{t-\tau_j(t)}^{t(t)} T(\xi)^\beta d\xi
\end{aligned}
$$

By putting in equation $S7$ and the previous stage duration equations we get:

$$
\phi(t-\tau_A(t)) = \phi(t) - \frac{1}{\alpha_A}
$$

for the adult stage and

$$
\phi(t-\tau_i(t)) = \phi(t) - \frac{1}{\gamma_i \alpha_I}
$$

for the immature stages, where i is E, L, or P. Also,

$$
\begin{aligned}
\phi(w_1) &= \phi(t-\tau_E(t)) \\
&= \phi(t) - \frac{1}{\gamma_E \alpha_I} \\
\phi(w_2) &= \phi(t-\tau_L(t)) \\
&= \phi(t) - \frac{1}{\gamma_L \alpha_I} \\
\phi(w_3) &= \phi(t-\tau_P(t)) \\
&= \phi(t) - \frac{1}{\gamma_P \alpha_I} \\
\phi(w_4) &= \phi(t-\tau_L(t)-\tau_E(t-\tau_L(t))) \\
&= \phi(t) - \frac{1}{\gamma_L \alpha_I} - \frac{1}{\gamma_E \alpha_I} \\
\phi(w_5) &= \phi(t-\tau_P(t)-\tau_L(t-\tau_P(t))) \\
&= \phi(t) - \frac{1}{\gamma_P \alpha_I} - \frac{1}{\gamma_L \alpha_I} \\
\phi(w_6) &= \phi(t-\tau_P(t)-\tau_L(t-\tau_P(t))-\tau_E(t-\tau_P(t)-\tau_L(t-\tau_P(t)))) \\
&= \phi(t) - \frac{1}{\gamma_P \alpha_I} - \frac{1}{\gamma_L \alpha_I} - \frac{1}{\gamma_E \alpha_I}
\end{aligned}
$$

Recall the survivorship function on the non-transformed time scale.

$$
S_i(t) = \exp\left(-\int_{t-\tau_i(t)}^{t} \delta_i(\xi)d\xi\right)
$$

In order to transform the survivorship functions, we will first introduce $\frac{h(t)}{h(t)}$ into the non-transformed survivorship function.

$$
\begin{aligned}
S_i(t) &= \exp\left(-\int_{t-\tau_i(t)}^{t} \delta_i(\xi)\frac{h(t)}{h(t)}d\xi\right) \\
&= \exp\left(-\int_{t-\tau_i(t)}^{t} \frac{\delta_i(\xi)}{h(t)}[h(t)d\xi]\right)
\end{aligned}
$$

From equation $S9$ we know that the term in the square braces is $d\phi$. This allows us to write the survivorship functions on the ϕ scale. Also, substituting in equation $S8$ gives us:

$$S_A(t) \quad = \quad \exp\left(-\int_{\phi-\frac{1}{\alpha_A}}^{\phi} \frac{\delta_A(x)}{m(x)}dx\right)$$

where $S_A(t)$ is the survivorship function for the adults and where

$$S_i(t) \quad = \quad \exp\left(-\int_{\phi-\frac{1}{\gamma_i\alpha_I}}^{\phi} \frac{\delta_i(x)}{m(x)}dx\right)$$

is the survivorship function for the immature stages, with i being equal to E, L, or P.

We can simplify by defining the delays as follows:

$$\begin{aligned}
\lambda_1 &= \frac{1}{\gamma_E\alpha_I} \\
\lambda_2 &= \frac{1}{\gamma_L\alpha_I} \\
\lambda_3 &= \frac{1}{\gamma_P\alpha_I} \\
\lambda_4 &= \frac{1}{\gamma_L\alpha_I} + \frac{1}{\gamma_E\alpha_I} \\
\lambda_5 &= \frac{1}{\gamma_P\alpha_I} + \frac{1}{\gamma_L\alpha_I} \\
\lambda_6 &= \frac{1}{\gamma_P\alpha_I} + \frac{1}{\gamma_L\alpha_I} + \frac{1}{\gamma_E\alpha_I}
\end{aligned}$$

So, putting this into our equations we get,

$$
\begin{aligned}
m(\phi)\frac{dL(\phi)}{d\phi} &= \rho A(\phi-\lambda_1)S_1(\phi)T(\phi)^{\beta} - \rho A(\phi-\lambda_4)S_2(\phi)T(\phi)^{\beta} \\
&\quad -\gamma_L\mu_3 \exp\left(\left(\frac{T(\phi)-\mu_4}{\mu_5}\right)^2 + \sigma_{exp}L(\phi)\right)L(\phi) \\
m(\phi)\frac{dA(\phi)}{d\phi} &= \rho A(\phi-\lambda_6)S_3(\phi)T(\phi)^{\beta} - \left(\mu_0 \exp\left(\left(\frac{T(\phi)-\mu_1}{\mu_2}\right)^4\right)A(\phi)\right)
\end{aligned}
$$

Recall that $T(\phi)^{\beta}$ is equal to $m(\phi)$; so if we divide through by $m(\phi)$ we get,

$$
\begin{aligned}
\frac{dL(\phi)}{d\phi} &= \rho A(\phi-\lambda_1)S_1(\phi) - \rho A(\phi-\lambda_4)S_2(\phi) - \frac{\gamma_L\mu_3}{m(\phi)} \exp\left(\left(\frac{T(\phi)-\mu_4}{\mu_5}\right)^2 + \sigma_{exp}L(\phi)\right)L(\phi) \\
\frac{dA(\phi)}{d\phi} &= \rho A(\phi-\lambda_6)S_3(\phi) - \left(\frac{\mu_0}{m(\phi)} \exp\left(\left(\frac{T(\phi)-\mu_1}{\mu_2}\right)^4\right)A(\phi)\right)
\end{aligned}
$$

where the survivorship equations in the fully transformed model are

$$
\begin{aligned}
S_1(\phi) &= \exp\left(-\gamma_E\mu_3\int_{\phi-\lambda_1}^{\phi}\frac{1}{m(x)}\exp\left(\left(\frac{T(x)-\mu_4}{\mu_5}\right)^2\right)dx\right) \\
S_2(\phi) &= \exp\left(-\gamma_E\mu_3\int_{\phi-\lambda_4}^{\phi-\lambda_2}\frac{1}{m(x)}\exp\left(\left(\frac{T(x)-\mu_4}{\mu_5}^2\right)\right)dx\right) \\
&\quad -\gamma_L\mu_3\int_{\phi-\lambda_2}^{\phi}\frac{1}{m(x)}\exp\left(\left(\frac{T(x)-\mu_4}{\mu_5}\right)^2 + \sigma_{exp}L(x)\right)dx\Bigg) \\
S_3(\phi) &= \exp\left(-\gamma_E\mu_3\int_{\phi-\lambda_6}^{\phi-\lambda_5}\frac{1}{m(x)}\exp\left(\left(\frac{T(x)-\mu_4}{\mu_5}\right)^2\right)dx\right. \\
&\quad -\gamma_L\mu_3\int_{\phi-\lambda_5}^{\phi-\lambda_3}\frac{1}{m(x)}\exp\left(\left(\frac{T(x)-\mu_4}{\mu_5}\right)^2 + \sigma_{exp}L(x)\right)dx \\
&\quad \left. -\gamma_P\mu_3\int_{\phi-\lambda_3}^{\phi}\frac{1}{m(x)}\exp\left(\left(\frac{T(x)-\mu_4}{\mu_5}\right)^2\right)dx\right).
\end{aligned}
$$
