# Supplementary material for: The Effect of Temperature on Anopheles Mosquito Population Dynamics and the Potential for Malaria Transmission
Source: PLoS One. 2013 Nov 14;8(11):e79276. doi: 10.1371/journal.pone.0079276 (PMC3828393; doi:10.1371/journal.pone.0079276)
Supplement: Text S3 — Model results when using the linear form of density-dependence. (PDF) [file pone.0079276.s021.pdf]

Text S3. Model results when using the linear form of density-dependence

We performed simulations on the model using the linear form of density-dependence (Figure S4) with constant temperatures from 16 to 40°C at one degree temperature increments. At all the temperatures explored, the model went to a fixed point equilibrium. As with the model using exponential density-dependent mortality, the mosquito populations were predicted to persist from 17 to 33°C. The adult equilibrium abundance was 2.0 to 4.7% the size of the larval equilibrium abundance. However, both the larval and adult populations reached much higher abundances in this version of the model. The peak in equilibrium abundance is shifted to the warmer end of the temperature range when compared to the predictions made by the model using exponential density-dependent mortality. The juvenile stages of the population impacted the equilibrium abundance of adults, making it more sensitive to temperature than would otherwise be predicted (Figure S5). The non-trivial equilibria were derived analytically and matched the equilibria found by numerical analysis.

The age structure of adult mosquitoes is an important determinant of the population vectorial capacity. The age structure of adults in our model was determined by adult mortality and by the dynamics in the juvenile stages. The model predicted that recruitment into the adult stage will generally increase as temperatures increase until it peaked at 31°C and began to decrease. The longevity of the adult population tended to increase also, peaking at 31°C then rapidly decreasing (Figure S6). The effects of larval density-dependent mortality are less pronounced in this version of the model and do not regulate the population abundance as much as in the version using exponential density-dependence. With the linear form of density-dependence the recruitment appears to follow the pattern of juvenile development rate except at the extreme ends of the temperature range.

The abundance of adult mosquitoes old enough to potentially transmit malaria at any given temperature was influenced by temperature and by the population structure predicted by the model. We used both the Detinova and the Paaijmans EIP prediction curves to calculate the population abundance of potentially infectious mosquitoes (Figure S7). These results were compared with the predictions when using the classical assumptions of constant mosquito abundance and temperature independence. When using the Detinova curve in combination with the linear density-dependent version of the model we found the potentially infectious population was predicted to increase with temperature peaking at 31°C and decreasing thereafter. The differences between the predictions made using the classic assumptions and our model results with Detinova prediction curve are most noticeable at the extremely high temper-

atures (32 to 40°C). The predictions from our model and the Detinova curve predicted a drop off in the population of mosquitoes that could potentially be infectious compared with the Detinova curve in combination with the classic assumptions. The peak at 31°C is two degrees warmer than the peak in larval equilibrium abundance. When using the Paaijmans prediction curve the model also predicted an increase in abundance as temperatures increase, however the peak and subsequent decline occur at the slightly cooler temperature of 30°C. When comparing the output from the predictions made using the classic assumptions and the model results with the Paaijmans curves, the differences were more subtle. The peak in potentially infectious mosquitoes using our model was predicted to be a bit narrower than the when using the classic assumptions. We also saw a decrease in the overall abundance when using our model; this was not seen when using the version of the model with exponential density-dependence.

We performed a local sensitivity analysis on the 12 parameters at each of the 25 temperatures explored for the version of the model using linear density-dependent mortality. We examined the influence that changes in each of the parameters had on several model outputs including larval and adult equilibrium abundance, the abundance of potentially infectious mosquitoes using both the Detinova and Paaijmans predictions, and the recruitment into the adult stage. All of the outputs were highly sensitive to juvenile development rate (α_I), larval density-dependent mortality (σ_{lin}) and the proportion of time spent in the larval stage (γ_L). Larval equilibrium abundance was also sensitive to two of the juvenile mortality parameters (μ_3 and μ_4), with the third (μ_5) becoming important at the low and high ends of the temperature range. Adult equilibrium abundance and recruitment into the adult stage were sensitive to the egg-laying rate (ρ) and were moderately sensitive to one of the adult mortality parameters (μ_0). Additionally at the low and high edges of the temperature range two juvenile mortality parameters (μ_4 and μ_5) became important. The abundance of potentially infectious mosquitoes were sensitive to changes in one adult mortality parameter (μ_0) particularly at cooler to mid-range temperatures and showed moderate sensitivity to egg-laying rate (ρ). Both of the predictions also showed sensitivity to two juvenile parameters ($\mu 4$ and μ_5) at the edges of the temperature range (Figure S8—S10, Table S1—S2).

The version of the model using the linear form of density-dependence in larval mortality predicted population peaks at higher temperatures than the version using exponential density-dependence. The population was also predicted to reach much higher abundances in general when compared with the exponential version. Population peaks predicted in the 30s°C are inconsistent with what is known about mosquitoes biologically.
